# The GSK-3β-FBXL21 Axis Contributes to Circadian TCAP Degradation and Skeletal Muscle Function

**DOI:** 10.1016/j.celrep.2020.108140

**Published:** 2020-09-15

**Authors:** Marvin Wirianto, Jiah Yang, Eunju Kim, Song Gao, Keshav Raj Paudel, Jong Min Choi, Jeehwan Choe, Gabrielle F. Gloston, Precious Ademoji, Randika Parakramaweera, Jianping Jin, Karyn A. Esser, Sung Yun Jung, Yong-Jian Geng, Hyun Kyoung Lee, Zheng Chen, Seung-Hee Yoo

**Affiliations:** 1 Department of Biochemistry and Molecular Biology, The University of Texas Health Science Center at Houston, 6431 Fannin St., Houston, TX 77030, USA; 2 Department of Internal Medicine, The University of Texas Health Science Center at Houston, 6431 Fannin St., Houston, TX 77030, USA; 3 Department of Biochemistry and Molecular Biology, Baylor College of Medicine, One Baylor Plaza, Houston, TX 77030, USA; 4 Department of Physiology and Functional Genomics, The University of Florida College of Medicine, Gainesville, FL 32610-0274, USA; 5 Department of Pediatrics, Baylor College of Medicine, Houston, TX 77030, USA; 6 Jan and Dan Duncan Neurological Research Institute, Texas Children’s Hospital, Houston, TX 77030, USA; 7 Lead Contact

## Abstract

FBXL21 is a clock-controlled E3 ligase modulating circadian periodicity via subcellular-specific CRYPTOCHROME degradation. How FBXL21 regulates tissue-specific circadian physiology and what mechanism operates upstream is poorly understood. Here we report the sarcomere component TCAP as a cytoplasmic substrate of FBXL21. FBXL21 interacts with TCAP in a circadian manner antiphasic to TCAP accumulation in skeletal muscle, and circadian TCAP oscillation is disrupted in *Psttm* mice with an *Fbxl21* hypomorph mutation. GSK-3β phosphorylates FBXL21 and TCAP to activate FBXL21-mediated, phosphodegron-dependent TCAP degradation. GSK-3β inhibition or knockdown diminishes FBXL21-Cul1 complex formation and delays FBXL21-mediated TCAP degradation. Finally, *Psttm* mice show significant skeletal muscle defects, including impaired fiber size, exercise tolerance, grip strength, and response to glucocorticoid-induced atrophy, in conjunction with cardiac dysfunction. These data highlight a circadian regulatory pathway where a GSK-3β-FBXL21 functional axis controls TCAP degradation via SCF complex formation and regulates skeletal muscle function.

## In Brief

Wirianto et al. find that the circadian E3 ligase FBXL21 drives rhythmic degradation of the sarcomeric protein TCAP in skeletal muscle. GSK-3β co-phosphorylates FBXL21 and TCAP and promotes SCF complex formation and phosphodegron-dependent TCAP turnover. Psttm mice, expressing a hypomorphic Fbxl21 mutant, show dysregulated TCAP degradation and impaired muscle function.

## Graphical Abstract

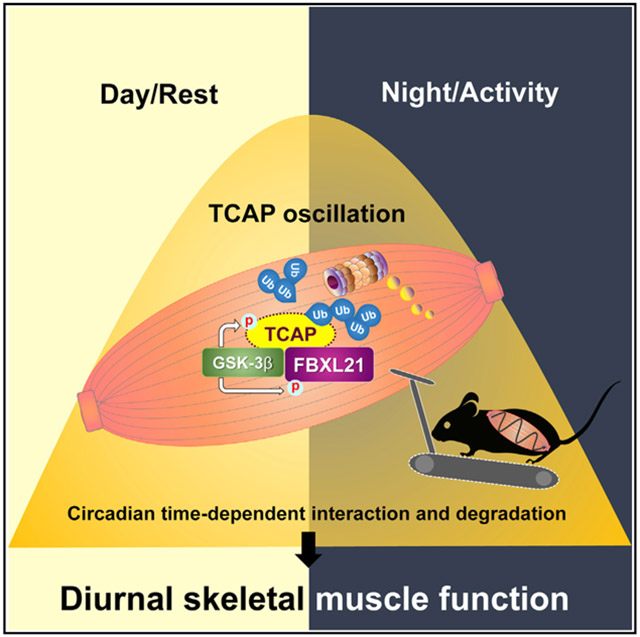

## INTRODUCTION

The mammalian circadian clock regulates tissue-specific gene expression to control metabolism, physiology, and behavior (Cederroth et al., 2019; Takahashi, 2017). The functional unit of the circadian clock is a cell-autonomous molecular oscillator composed of auto-regulatory transcriptional-translational feedback loops of clock genes encoding positive (CLOCK, NPAS2, BMAL1, and RORs) and negative (PER1/2, CRY1/2, and REVERBs) components (Takahashi, 2017). The oscillator is governed by a complex mechanism encompassing transcriptional, post-transcriptional, and post-translational regulatory steps (Gallego and Virshup, 2007; Green, 2018). In particular, ubiquitination-mediated proteasomal degradation is a critical post-translational mechanism in circadian oscillation, controlling clock protein degradation at the end of a circadian cycle prior to starting a new one (Stojkovic et al., 2014). The ubiquitination cascade involves serial enzymatic reactions by an E1 ubiquitin-activating enzyme, an E2 ubiquitin-conjugating enzyme, and an E3 ubiquitin ligase (Hershko et al., 1983). E3 ubiquitin ligases recognize and attach ubiquitin (Ub) to substrate proteins; therefore, they are mainly responsible for target specificity and downstream processes. Various E3 ligases and deubiquitinases regulate degradation of core clock proteins (Gallego and Virshup, 2007; Stojkovic et al., 2014), although their functions and regulatory mechanisms in circadian physiology are not well understood.

F box E3 ligases are characterized by F box domains spanning ~50 amino acids. F box domains mediate formation of SCF Ub-ligase complexes, each containing Skp1, Cullin, and F box proteins, for Ub-mediated proteolysis (Kipreos and Pagano, 2000). F box proteins are known to regulate circadian rhythmicity in various model systems (Busino et al., 2007; Dardente et al., 2008; Eide et al., 2005; Godinho et al., 2007; Grima et al., 2002; Harmon and Kay, 2003; He et al., 2003; Hirano et al., 2013; Imaizumi et al., 2003; Ko et al., 2002; Koh et al., 2006; Shirogane et al., 2005; Siepka et al., 2007; Somers et al., 2004; Yoo et al., 2013). Previous reports illustrate functional and mechanistic interplay between paralogous E3 ligases (Adler et al., 2017; Fuchs et al., 2004; Scott et al., 2016; Siepka et al., 2007; Yoo et al., 2013). Although β-TRCP1 and β-TRCP2 appear to be largely redundant in substrate recognition and degradation (Fuchs et al., 2004), we and others uncovered an unexpected functional antagonism between the circadian E3 ligases F-box/LRR-repeat protein 3 and 21 (FBXL3 and FBXL21). Hypomorphic Fbxl3 mutations were initially found to lengthen the circadian period and stabilize CRY proteins by attenuating SCF-FBXL3-mediated proteasomal degradation (Busino et al., 2007; Godinho et al., 2007; Siepka et al., 2007). More recently, we reported a short-period circadian mutant, *Past-time* (*Psttm*; indicating the homozygous mutant *Fbxl21^Psttm/Psttm^*), where the mutation was mapped to a gene encoding FBXL21, a homolog of FBXL3 (Yoo et al., 2013). Unexpectedly, the reduced level and activity of FBXL21 in *Psttm* led to destabilization of CRY proteins. Mechanistic studies revealed an unusual dual function of FBXL21. On one hand, FBXL21 forms an SCF E3 ligase complex that slowly degrades CRY in the cytoplasm, and on the other hand, it antagonizes the stronger E3 ligase activity of FBXL3 in the nucleus by sequestering CRY from FBXL3. *Fbxl21* is also a clock-controlled gene (Dardente et al., 2008); its transcript and protein levels exhibit circadian oscillation (Yoo et al., 2013). Another study also described antagonism of FBXL3 and FBXL21 for CRY degradation but proposed a mechanism involving heterogeneous ubiquitination chains (Hirano et al., 2013). Identification of additional protein substrates will provide mechanistic insights and reveal how these FBXLs regulate circadian physiology.

Regulation of F box proteins could impinge on multiple steps, including synthesis, dimerization, degradation, and SCF activity (Lee and Diehl, 2014; Skaar et al., 2013; Wang et al., 2014; Welcker et al., 2013). Particularly, neddylation at the C terminus of CULLIN1 induces structural rearrangement in its backbone, increasing SCF E3 ligase activity (Lydeard et al., 2013). In contrast to the molecular mechanisms that regulate F box protein function, upstream cellular signaling for most F box proteins in SCF complex formation and E3 ligase activation remains largely unclear. Studies have indicated a role of post-translational modifications (PTMs), including sumoylation and particularly phosphorylation, in altering the subcellular localization and E3 ligase activity of F box proteins (Fuchs et al., 2002; Gao et al., 2009; Jura et al., 2006; Lin et al., 2009; Wang et al., 2018; Xu et al., 2010). How FBXL21 activity is regulated upstream is largely unknown.

To identify additional cellular targets and regulatory mechanisms of FBXL21, we conducted a yeast 2-hybrid (Y2H) screen and found that Telethonin, also known as Titin cap or TCAP (Valle et al., 1997), is a cytoplasmic substrate of FBXL21. Mechanistic studies revealed circadian regulation of TCAP ubiquitination by FBXL21 and uncovered a nodal regulatory function of the GSK-3β-FBXL21 axis in SCF complex formation, substrate turnover, and muscle function.

## RESULTS

### Identification of TCAP as a Cytoplasmic Substrate of FBXL21

**Figure 1.**
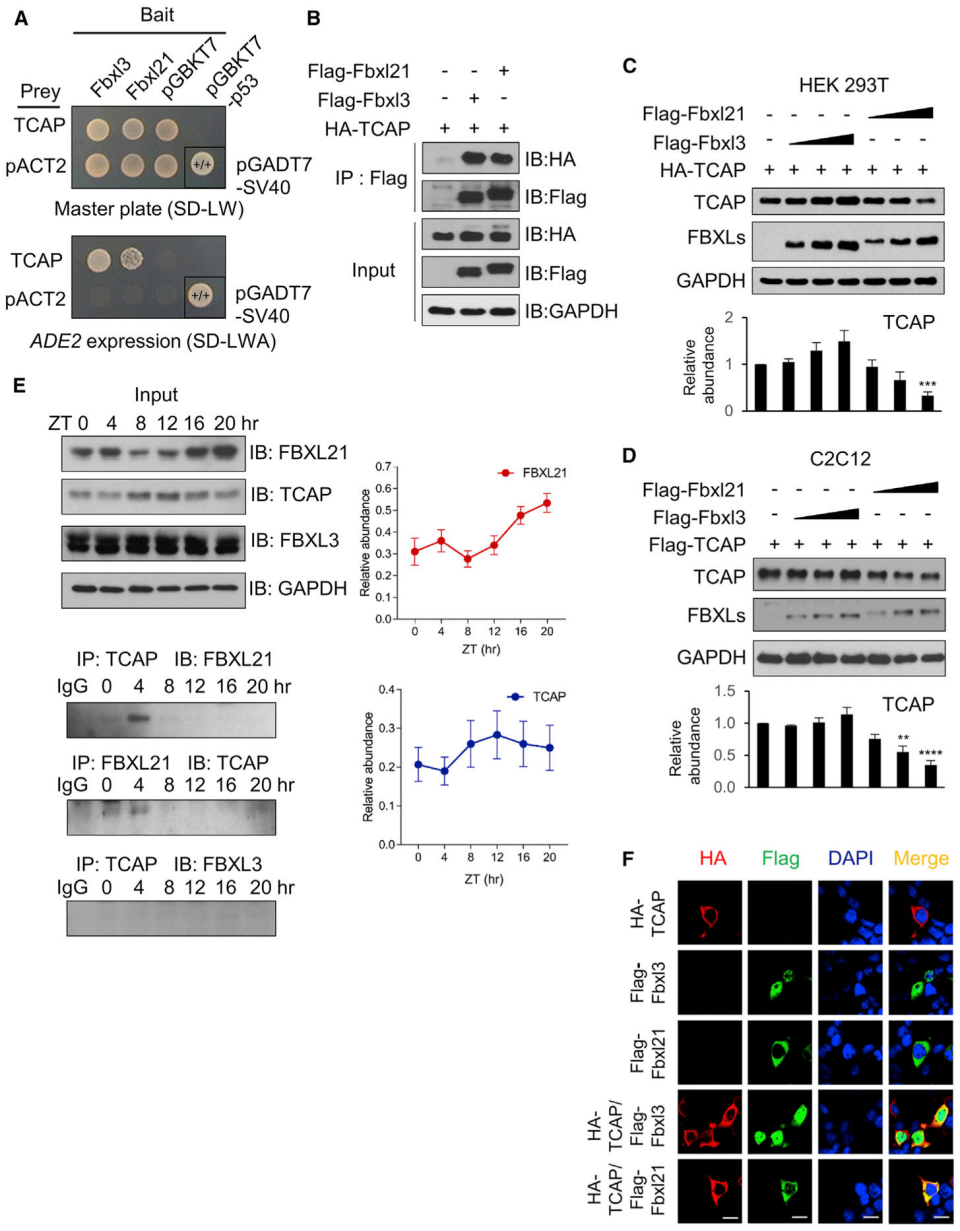
Identification of TCAP as a FBXL21 Substrate (A) Y2H test showing that FBXL3 and FBXL21 interact with TCAP. Positive control yeasts were transformed with the pGBKT7-p53 bait plasmid and the pGADT7-SV40 Tag prey plasmid. pGBKT7-53 encodes the Gal4 DNA binding domain (BD) fused with murine p53; pGADT7-T encodes the Gal4 activation domain (AD) fused with SV40 large T antigen. (B) Interaction of FBXL3 and FBXL21 with TCAP. 293T cells were transfected with FLAG-Fbxl3, FLAG-Fbxl21, and hemagglutinin (HA)-TCAP, and immunoprecipitation was performed using anti-FLAG antibody (M2). (C and D) FBXL21, but not FBXL3, decreases the TCAP amount in a dose-dependent manner. 293T (C) and C2C12 (D) cells were co-transfected with the indicated constructs. The graphs below show quantification of the dose effects of FBXL3 and FBXL21 on TCAP stability using unnormalized optical density (OD) values, and error bars represent ± SEM (n = 4). TCAP abundance shows a significant statistical difference between TCAP alone and different FBXL21 concentrations in 293T and C2C12 cells (t test, **p < 0.01, ***p < 0.001, ****p < 0.0001). (E) Top left panel: FBXL21 showed anti-phase oscillation with TCAP in skeletal muscle. Right panels: quantification of FBXL21 and TCAP levels using unnormalized OD values. Error bars represent ± SEM (n = 3). Two-way ANOVA shows a significant statistical difference between TCAP and FBXL21 (p < 0.0001). Bonferroni’s multiple comparisons test shows a significant difference at ZT16 (p < 0.05) and ZT20 (p < 0.01). One-way ANOVA with Tukey’s post hoc analysis shows a statistically significant differences in the FBXL21 amount between time points (*p < 0.05). Bottom left panel: co-immunoprecipitation showing circadian time-dependent TCAP-FBXL21 interaction. (F) Differential localization of FBXL3 and FBXL21 in 293T cells. Scale bars, 20 μm. See also Figure S1.

To further dissect the cellular mechanisms of FBXL21, we performed a Y2H screen for putative target substrates using a human skeletal muscle expression library, given the important role of the clock in skeletal muscle (Harfmann et al., 2015). The established substrate CRY2 was used as a positive control (Figure S1A). TCAP, a core sarcomeric component capping the titin proteins, was identified as a positive hit (Figure 1A). TCAP is a small (19 kDa), highly abundant cytoplasmic protein expressed exclusively in skeletal muscle and the heart (Valle et al., 1997). TCAP interacts with titin through its N-terminal β sheet to anchor titin to the Z-disc (Gregorio et al., 1998; Mues et al., 1998; Pinotsis et al., 2006), and TCAP deficiency in mice and humans causes dysfunction in heart and skeletal muscle. Interestingly, TCAP mRNA and its protein product display robust circadian expression (Podobed et al., 2014). To validate TCAP as a possible circadian substrate of FBXLs, we co-expressed TCAP with FBXL21 and FBXL3 in 293T cells and performed co-immunoprecipitation. FBXL21 and FBXL3 were found to bind to TCAP (Figure 1B). However, ectopic expression of FBXL21, but not FBXL3, dose-dependently destabilized TCAP in 293T and C2C12 cells, suggesting that FBXL21 functions as the primary E3 ligase for cytoplasmic TCAP proteins (Figures 1C and 1D).

To define *in vivo* interaction between TCAP and FBXLs, we performed co-immunoprecipitation using gastrocnemius muscles collected from wild-type (WT) C57BL/6 mice during one 12 h:12 h light:dark (LD) circadian cycle every 4 h at the indicated zeitgeber time (ZT). Although FBXL3 did not show any significant interaction with TCAP throughout the circadian cycle, presumably because of the distinct localization of TCAP (strictly cytoplasmic; Valle et al., 1997) and FBXL3 (predominantly nuclear; Siepka et al., 2007), FBXL21 and TCAP displayed circadian oscillation of binding, peaking at ZT0–ZT4 (Figure 1E).

Consistent with TCAP as a sarcomere component, ectopic expression of TCAP showed cytoplasmic accumulation in 293T cells (Figure 1F). When expressed alone, FBXL3 and FBXL21 were mainly localized in the nucleus and the cytoplasm, respectively, as we reported previously (Figure 1F; Yoo et al., 2013). Co-expression of TCAP led to FBXL3 distribution in the nucleus and cytoplasm (Figure 1F), perhaps because their interaction did not cause TCAP turnover (Figures 1C and 1D). FBXL21 and TCAP co-localized in the cytoplasm, further suggesting a role of FBXL21 in TCAP degradation.

### FBXL21 Directly Regulates TCAP Degradation by F Box-Dependent Ubiquitination

**Figure 2.**
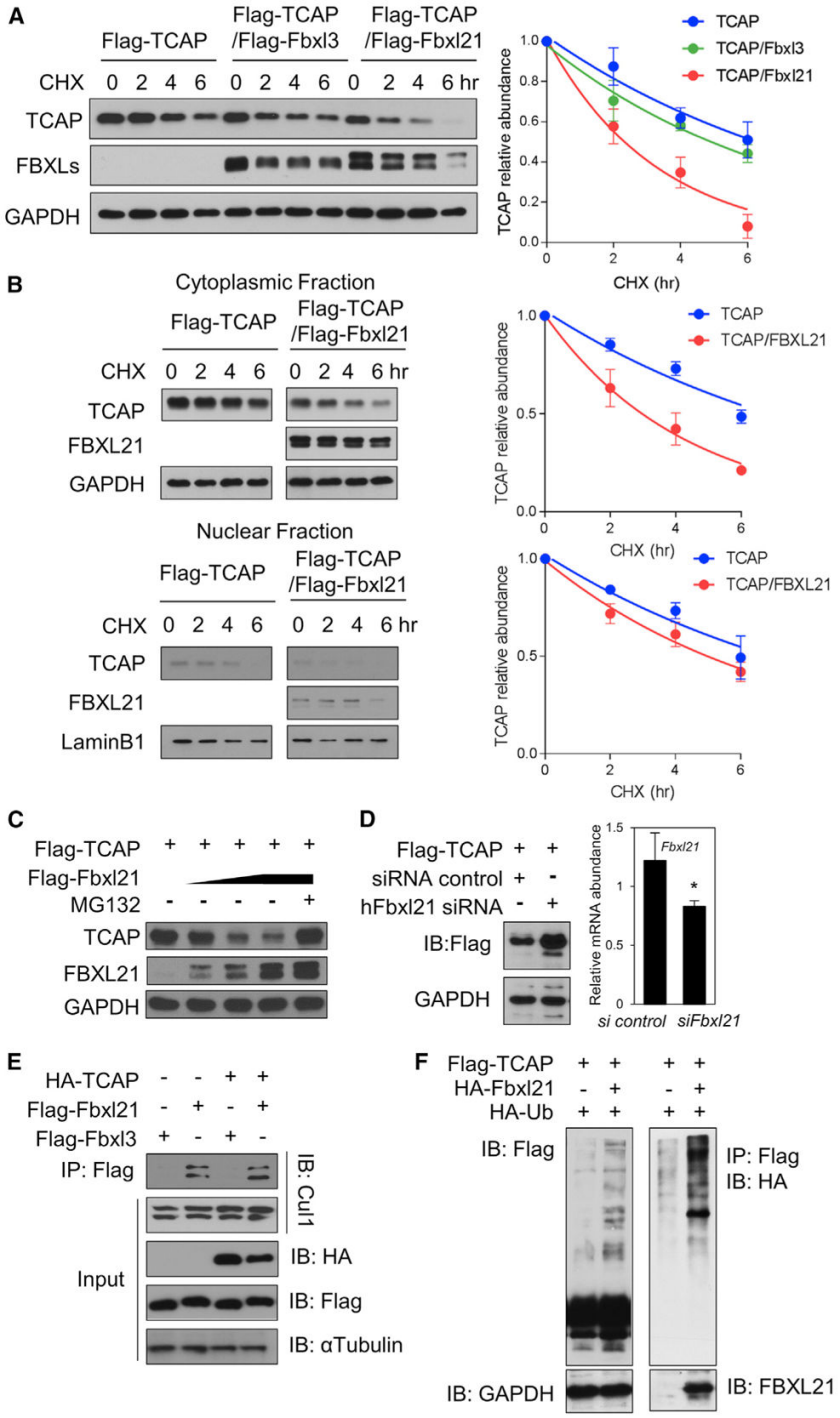
FBXL21 Mediates TCAP Proteasomal Degradation by Ubiquitination (A) 293T cells were co-transfected with the indicated constructs. Thirty-two hours after transfection, cells were treated with 100 μg/mL CHX and incubated for the indicated time before harvest. Immunoblotting was performed to detect TCAP (~25 kD) and FBXLs using anti-FLAG antibody. Right panel: quantification of the effect of FBXL3 and FBXL21 on TCAP stability. Error bars represent ± SEM (n = 3). Half-life was determined by using nonlinear, one-phase decay analysis (TCAP, 6.1 h, TCAP/FBXL3, 5.0 h, TCAP/FBXL21, 2.3 h; the half-life parameter, K, is significantly different in Fbxl21 co-expression: p < 0.0001). (B) Accelerated cytoplasmic TCAP degradation by FBXL21 in the cytoplasm. Immunoblotting was performed using cytoplasmic and nuclear fractions with anti-FLAG antibody. Right panels: quantification of cytoplasmic TCAP and nuclear TCAP. Error bars represent mean ± SEM (n = 3). Half-life was determined by using nonlinear, one-phase decay analysis (CytoTCAP, 6.5 h; CytoTCAP/FBXL21, 3.0 h, NuTCAP, 6.7 h; NuTCAP/FBXL21, 5.0 h; the half-life parameter, K, is significantly different with Fbxl21 co-expression: p < 0.0001). (C) MG132 inhibited TCAP degradation by FBXL21. (D) Knockdown of Fbxl21 increased TCAP expression in 293T cells. Right panel: real-time RT-PCR analysis of hFbxl21 mRNA expression (*p < 0.05). (E) FBXL21-Cullin-1 complex formation in the cytoplasm. (F) TCAP ubiquitination by FBXL21. See also Figure S1.

Next we performed cycloheximide (CHX) chase assays to measure the effect of FBXLs on TCAP turnover. FBXL21 accelerated TCAP degradation in 293T cells and C2C12 myoblast cells, whereas FBXL3 activity was much less significant (Figures 2A and S1B). For example, the TCAP half-life was estimated to be 6.1 h by itself and 5.0 h and 2.3 h with Fbxl3 and Fbxl21 co-transfection, respectively, in 293T cells. We next compared the rates of degradation and found that FBXL21 expression significantly increased the TCAP degradation rate in the cytoplasm but not in the nucleus (Figure 2B).

To ascertain whether FBXL21-mediated TCAP degradation is mediated by ubiquitination-dependent proteasomal degradation, we treated 293T cells with the 26S proteasome inhibitor MG132 and observed inhibition of dose-dependent TCAP degradation by FBXL21 (Figure 2C). Knockdown of endogenous Fbxl21 in 293T cells by hFbxl21 small interfering RNA (siRNA) transfection increased TCAP accumulation compared with control siRNA, suggesting that endogenous FBXL21 regulates FLAG-TCAP degradation (Figure 2D). Next we investigated whether TCAP binding can modulate subcellular FBXL21-CULLIN1 complex formation by co-immunoprecipitation. Previously, binding of the substrate CRY1 to FBXL3 was found to promote SCF complex formation (Yumimoto et al., 2013). In contrast, FBXL21 formed active SCF E3 ligase complexes in the cytoplasm, and TCAP ectopic expression did not increase neddylated-CULLIN1-FBXL21 complex formation, which is indicative of active E3 ligase activity (Figure 2E).

Next we performed ubiquitination assays by expressing TCAP, FBXL21, and Ub in 293T cells in the presence of MG132 (Figure 2F). FBXL21 co-expression enriched poly-ubiquitinated TCAP compared with TCAP by itself, suggesting that FBXL21 mediates TCAP ubiquitination (Figure 2F). MDM2 has been reported to promote TCAP proteasomal degradation via a mechanism independent of its ring domain (Tian et al., 2006). In contrast, loss-of-function mutant FBXL21 with F box deletion (Yoo et al., 2013) did not promote formation of poly-ubiquitinated TCAP compared with the WT (Figure S1C). These results suggest that, unlike MDM2 (Tian et al., 2006), FBXL21-mediated TCAP ubiquitination entails direct Ub transfer from the SCF E3 ligase complex containing FBXL21.

### Identification of TCAP Ubiquitination Lysine Sites by FBXL21

**Figure 3.**
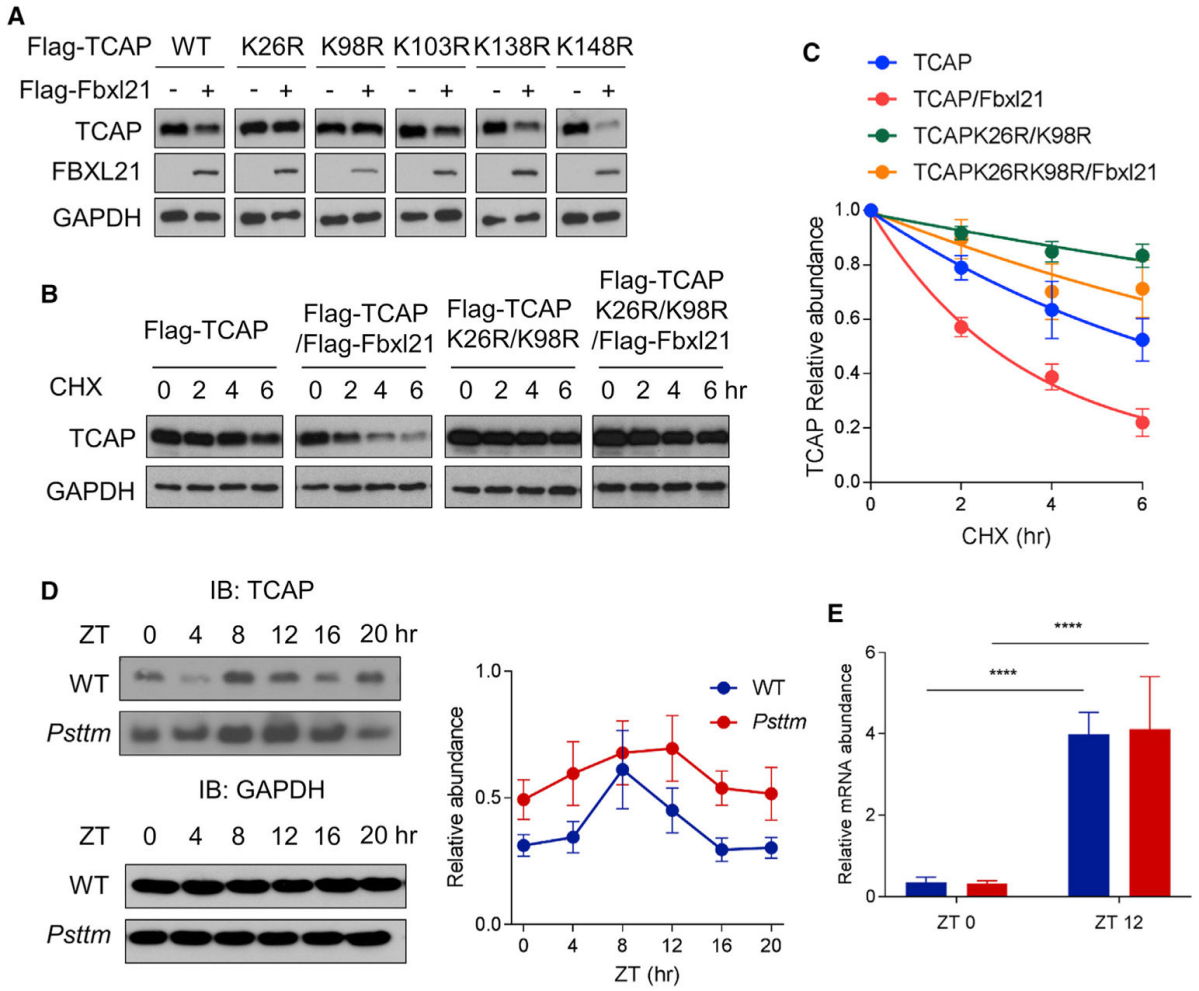
Identification of TCAP Ubiquitination Sites by FBXL21 and Altered TCAP Circadian Oscillation in *Psttm* Skeletal Muscle (A) The five lysine sites in TCAP were individually mutated to arginine, and the stability of each K-to-R mutant was examined by co-transfecting the Fbxl21 expression construct into 293T cells. (B) Impaired FBXL21-mediated degradation of the TCAP K26R/K98R mutant. Immunoblotting was performed to detect TCAP (~25 kD) and FBXLs using anti-FLAG antibody. (C) Quantification of the effect of FBXL21 on WT TCAP and K26R/K98R stability. Error bars represent ± SEM (n = 3). Half-life: TCAP, 5.5 h, TCAP/FBXL21, 2.3 h, TCAPK26R/K98R, 19.4 h, TCAPK26R/K98R /FBXL21, 9.4 h). (D) TCAP oscillation in skeletal muscle from WT and *Psttm* mutant mice. Immunoblotting was performed using total protein extracts with TCAP antibody. Representative blots from three independent experiments are shown, and quantification is shown in the right panel (error bars represent ± SEM, n = 3). One-way ANOVA with Tukey’s post hoc analysis shows statistically significant differences in the TCAP amount between time points in WT (*p < 0.05) but not *Psttm* mice. Two-way ANOVA shows significant statistical differences between WT and *Psttm* mice for TCAP expression (p < 0.01). Blue and red circles represent WT and *Psttm* mice, respectively. (E) Real-time RT-PCR analysis of TCAP mRNA expression in WT (blue) and *Psttm* (red) mice. Two-way ANOVA shows a significant statistical difference for TCAP mRNA at ZT0 and ZT12 (error bars represent ± SEM, n = 3; ****p < 0.0001) but no significance between WT and *Psttm* mice. See also Figure S2.

To identify the ubiquitination sites required for TCAP degradation, the five lysines in TCAP were converted individually to arginine. In an initial endpoint degradation assay with FBXL21 co-expression, the K26R and K98R mutations conferred resistance to FBXL21-mediated TCAP degradation (Figure 3A). Therefore, we generated a K26R/K98R double mutant and performed CHX degradation assays in 293T cells. As shown in Figure 3B, the K26R/K98R double mutant displayed a significantly reduced degradation rate with FBXL21 co-expression relative to the WT TCAP (Figure 3C), indicating that FBXL21 targets K26 and K98 for ubiquitination-mediated proteasomal degradation of TCAP.

### The *Psttm* Mutation Disrupted TCAP Protein Circadian Oscillation

We examined the effect of the *Fbxl21* hypomorph mutation (*Psttm*, G149E) on TCAP circadian oscillation in skeletal muscle and heart tissues. TCAP protein showed circadian oscillation in WT tissues (Figures 3D and S2A); however, TCAP amounts were significantly elevated and circadian oscillation was disrupted in *Psttm* mouse tissues, whereas BMAL1 levels were largely unaltered (Figure S2B).

We next performed qPCR to examine TCAP mRNA levels in WT and *Psttm* skeletal muscle at ZT0 and ZT12, corresponding to trough and peak points during its oscillation. The TCAP mRNA level was markedly higher at ZT12 compared with ZT0 but not significantly different between the WT and *Psttm* (Figure 3E), suggesting that the increase in TCAP protein amount in *Psttm* skeletal muscle is primarily due to impaired protein degradation. These results strongly suggest that *Fbxl21* functional impairment leads to TCAP protein stabilization. Of note, the TCAP protein level peaked at ZT8 in the WT (Figure 3D), a time when its interaction with FBXL21 was largely abrogated (Figure 1E). This result further indicates that FBXL21-mediated circadian proteasomal degradation of TCAP contributes to TCAP protein oscillation in addition to the rhythmic *Tcap* transcript levels, as reported previously (Hodge et al., 2019; Podobed et al., 2014).

### FBXL21 E3 Ligase Activity for TCAP Is Regulated by GSK-3β

**Figure 4.**
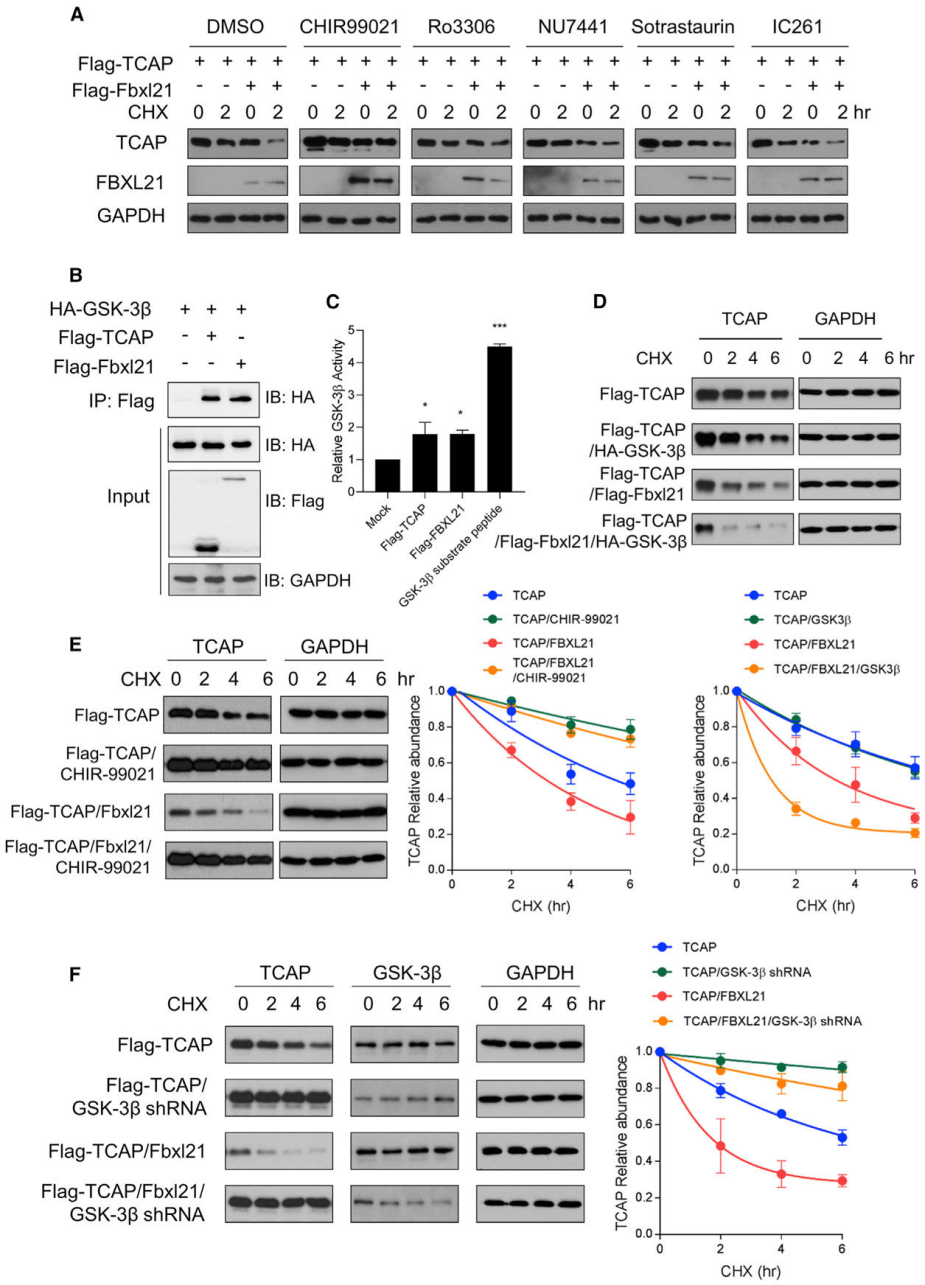
GSK-3β Regulates FBXL21-Mediated TCAP Degradation (A) 293T cells were co-transfected with the indicated constructs. Twelve hours after transfection, cells were treated with the indicated kinase inhibitors for 24 h before CHX treatment. (B) Interaction of TCAP and FBXL21 with GSK-3β. 293T cells were transfected with FLAG-TCAP, FLAG-Fbxl21, and HA-GSK-3β, and immunoprecipitation was performed using anti-FLAG antibody (M2). (C) GSK-3β *in vitro* kinase assay. Affinity-purified FLAG-TCAP and FLAG-FBXL21 from 293T cells were used as kinase reaction substrates. One-way ANOVA shows a significant statistical difference between mock (pCMV-3XFLAG empty vector) and FLAG-TCAP and FLAG-FBXL21 (*p < 0.05) and GSK-3β (***p < 0.001, n = 3). (D) GSK-3β ectopic expression accelerated TCAP degradation in an FBXL21-dependent manner. Bottom panel: quantification of the effect of ectopic expression of GSK-3β on TCAP stability. Error bars represent ± SEM (n = 3). Half-life: TCAP, 5.5 h; TCAP/GSK-3β, 5.2 h; TCAP/FBXL21, 2.3 h; TCAP/FBXL21/GSK-3β, 0.8 h. (E) The GSK-3 inhibitor CHIR-99021 decelerated FBXL21-mediated TCAP degradation. Right panel: quantification of the effect of CHIR-99021 on TCAP stability. Error bars represent ± SEM (n = 3). Half-life: TCAP, 5.3 h; TCAP/CHIR-99021, 15.9 h; TCAP/FBXL21, 3.1 h; TCAP/FBXL21/CHIR, 12.3 h. (F) GSK-3β knockdown decelerated FBXL21-mediated TCAP degradation. Right panel: quantification of the effect of GSK-3β knockdown on TCAP stability. Error bars represent ± SEM (n = 3). Half-life: TCAP, 4.1 h; TCAP/GSK-3β shRNA, 32.0 h; TCAP/FBXL21, 1.1 h; TCAP/FBXL21/GSK-3β shRNA, 12.9 h.

We next investigated the upstream regulatory mechanism for FBXL21 activity. Given the reported regulatory phosphorylation events in both substrates (phosphodegron) and E3 ligases (Filipčik et al., 2017; Gao et al., 2009; Kazlauskaite et al., 2014; Lin et al., 2009), we searched putative phosphorylation sites of FBXL21 (phosphoScan, NetPhos3.1; Blom et al., 2004) and identified 5 candidate kinases with high scores (>0.45), including GSK-3, DNA-PK, CDK, protein kinase C (PKC), and CK1. Using specific inhibitors (CHIR-99021, GSK-3; NU7441, DNA-PK; Ro3306, CDK; Sotrastaurin, PKC; IC261, CK1), protein degradation assays showed that the GSK-3 inhibitor CHIR-99021 strongly repressed FBXL21-mediated TCAP degradation (Figure 4A). Because GSK-3β, but not GSK-3α, plays a crucial role in muscle (Beurel et al., 2015; van der Velden et al., 2008; Verhees et al., 2011), we focused on GSK-3β for muscle-specific protein TCAP degradation.

To determine whether GSK-3β targets FBXL21 and/or TCAP, we co-expressed GSK-3β with FBXL21 or TCAP in 293T cells and performed co-immunoprecipitation. Interestingly, TCAP and FBXL21 were found to bind to GSK-3β (Figure 4B), suggesting that both could be GSK-3β substrates. We therefore conducted *in vitro* kinase assays using affinity purified FLAG-TCAP and FLAG-FBXL21, which showed that TCAP and FBXL21 were efficiently phosphorylated by GSK-3β (Figure 4C). Furthermore, ectopic expression of GSK-3β accelerated TCAP degradation in a FBXL21-dependent manner (Figure 4D), providing gain-of-function evidence of a role of FBXL21 in GSK-3β-regulated TCAP degradation. In TCAP degradation assays using 293T cells, FBXL21-mediated TCAP degradation was significantly retarded by CHIR-99021 (Figure 4E) or co-transfection of GSK-3β shRNA (Figure 4F). These results demonstrate that GSK-3β phosphorylates FBXL21 and TCAP and plays a regulatory role in FBXL21-mediated TCAP ubiquitination.

### Distinct Functional Mechanisms of GSK-3β-Mediated Phosphorylation for TCAP and FBXL21

**Figure 5.**
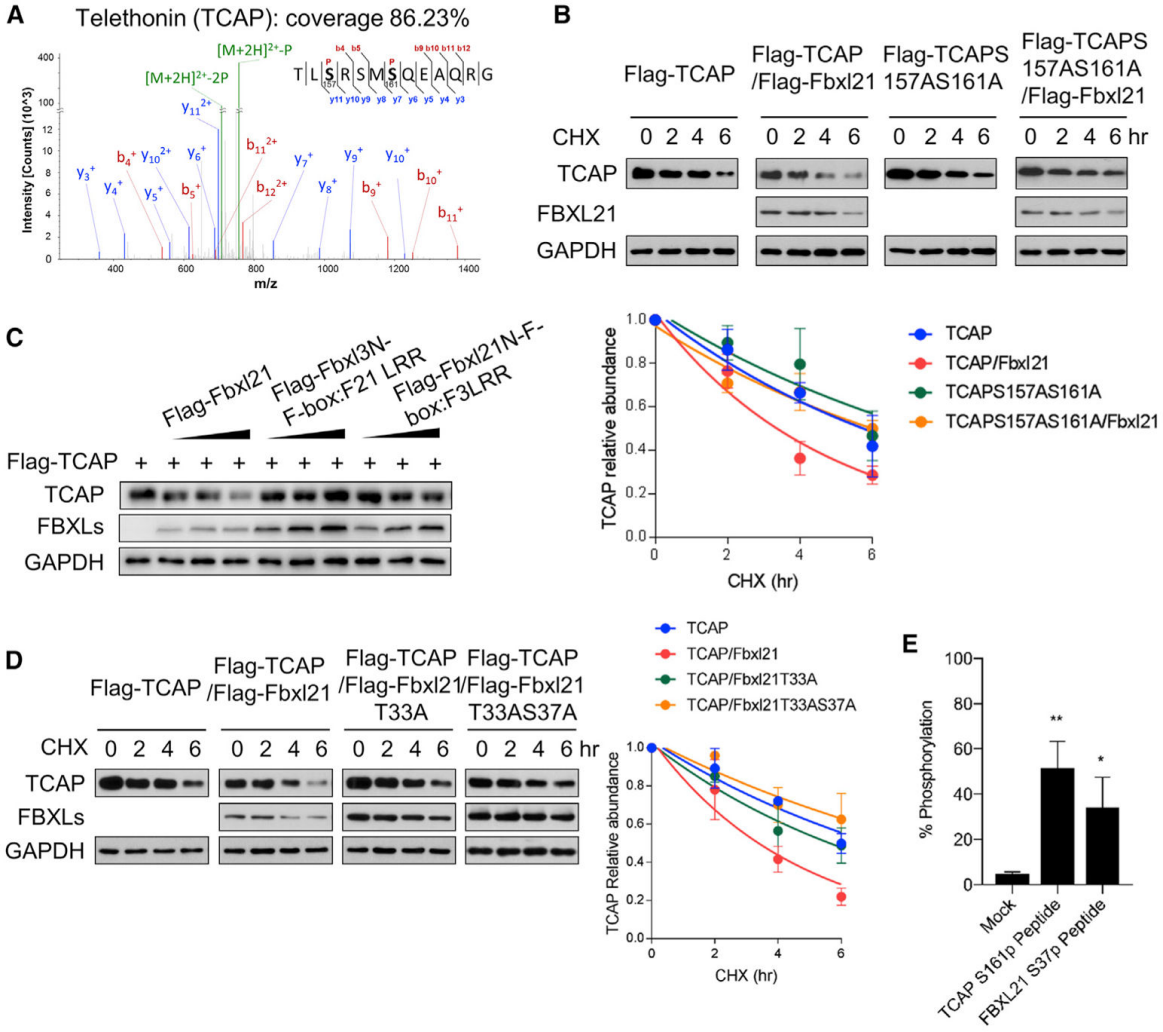
Identification of Phosphodegron Sites for TCAP and FBXL21 E3 Ligase Activity Regulation Sites (A) Phosphorylated peptides, including S157 and S161, of TCAP were identified with high confidence by MS. (B) The TCAP S157AS161A mutant showed decelerated degradation compared with WT TCAP. Bottom panel: quantification of the effect of FBXL21 on WT TCAP and TCAP S157AS161A stability. Error bars represent ± SEM (n = 3). Half-life: TCAP, 5.5 h; TCAP/FBXL21, 3.2 h; TCAP S157AS161A, 6.8 h; TCAP S157AS161A/FBXL21, 6.2 h. (C) 293T cells were co-transfected with constructs expressing WT and chimeric FBXL21 (Figure S6A). (D) The FBXL21 T33A and FBXL21 T33AS37A mutants showed reduced E3 ligase activity for TCAP. Right panel: quantification of TCAP degradation. Error bars represent ± SEM (n = 3). Half-life: TCAP, 6.6 h; TCAP/FBXL21, 3.1 h; TCAP/FBXL21T33A, 5.4 h; TCAP/FBXL21T33AS37A, 8.3 h; the half-life parameter, K, is significantly different. (E) GSK-3β *in vitro* kinase assay. TCAP and FBXL21 GSK-3β target site peptides were used as kinase reaction substrates. Error bars represent ± SEM (n = 3). One-way ANOVA shows significant statistical difference of the percentage of phosphorylation between mock (no substrate control) and the TCAP S161p peptide (**p < 0.01) and the FBXL21 S37p peptide (*p < 0.05). See also Figures S3 and S4.

To identify the target phosphorylation sites of GSK-3β for TCAP and FBXL21, we performed mass spectrometry. FLAG-tagged TCAP and FBXL21 proteins were isolated by affinity purification and analyzed by liquid chromatography-tandem mass spectrometry (LC-MS/MS) to determine phosphorylated sites. We identified S157 and S161 phosphorylation in TCAP corresponding to GSK-3β consensus phosphorylation sites (SXXXS) (Figure 5A). S157/S161 have been reported previously as protein kinase D (PKD) phosphorylation sites in cardiac myocytes and to be involved in t-tubule organization (Candasamy et al., 2014); however, TCAP phosphorylation was not affected by PKD inhibition (Candasamy et al., 2014). To determine whether these sites function as a phosphodegron for TCAP degradation by FBXL21, we mutated both serine residues to alanine and performed CHX half-life measurement of the TCAP S157A/S161A mutant. The TCAP S157A/S161A mutant showed significantly retarded FBXL21-mediated degradation, suggesting that these two sites constitute a phosphodegron for ubiquitination-mediated degradation by FBXL21 (Figure 5B).

Because of the size similarity between FLAG-tagged FBXL21 and immunoglobulin G (IgG) heavy chain, we were not able to isolate single bands from affinity purification. Therefore, we generated chimeric FBXLs (Figure S3A) to narrow down functional domains with E3 ligase activity for TCAP. All chimeric FBXLs were found to bind to TCAP (Figure S3B). Compared with WT FBXL21, moderately reduced but dose-dependent TCAP degradation activity was observed from a chimeric protein containing the FBXL21 N terminus (Figure 5C). In comparison, a chimeric protein containing the FBXL3 N terminus did not show TCAP degradation activity, suggesting a regulatory domain in the N terminus of FBXL21 and not in the LRR (leucine-rich repeat). In this N-terminal region, we found T33 as a candidate GSK-3β target site based on its consensus sequence. We therefore performed site-directed mutagenesis of T33 and S37 (candidate target and priming sites for GSK-3β). The T33A single and T33A/S37A double mutants showed significant deficits in TCAP degradation assays (Figure 5D), although their binding to TCAP was not compromised (Figure S3C). Furthermore, *in vitro* kinase assays showed that GSK-3β efficiently transferred phosphate groups to TCAP S157 and FBXL21 T33 peptides (Figure 5E). These results show direct GSK-3β phosphorylation of TCAP S157 and FBXL21 T33 sites.

To investigate the GSK-3β role in circadian TCAP dynamics, we employed differentiated C2C12 cells (Zhang et al., 2012). First we determined whether FBXL21 and TCAP are endogenously expressed in differentiated C2C12 cells. On differentiation day 2 after serum starvation, TCAP (Langley et al., 2002; Markert et al., 2008) and FBXL21 were found to be expressed in C2C12 cells, and expression persisted until day 5 (Figure S4A). Next we used the GSK-3 inhibitors CHIR-99021 and lithium to examine effects of FBXL21 inhibition on TCAP circadian oscillation. Importantly, consistent with the skeletal muscle tissue results (Figure 1E), we observed antiphasic circadian oscillations of TCAP and FBXL21, which was abolished by GSK-3β inhibitor treatment (Figure S4B). These results provide functional evidence that GSK-3β strongly regulates the reciprocal circadian oscillations of TCAP accumulation and FBXL21 activity.

### GSK-3β Regulates FBXL21-TCAP and FBXL21-SCF Complex Formation

**Figure 6.**
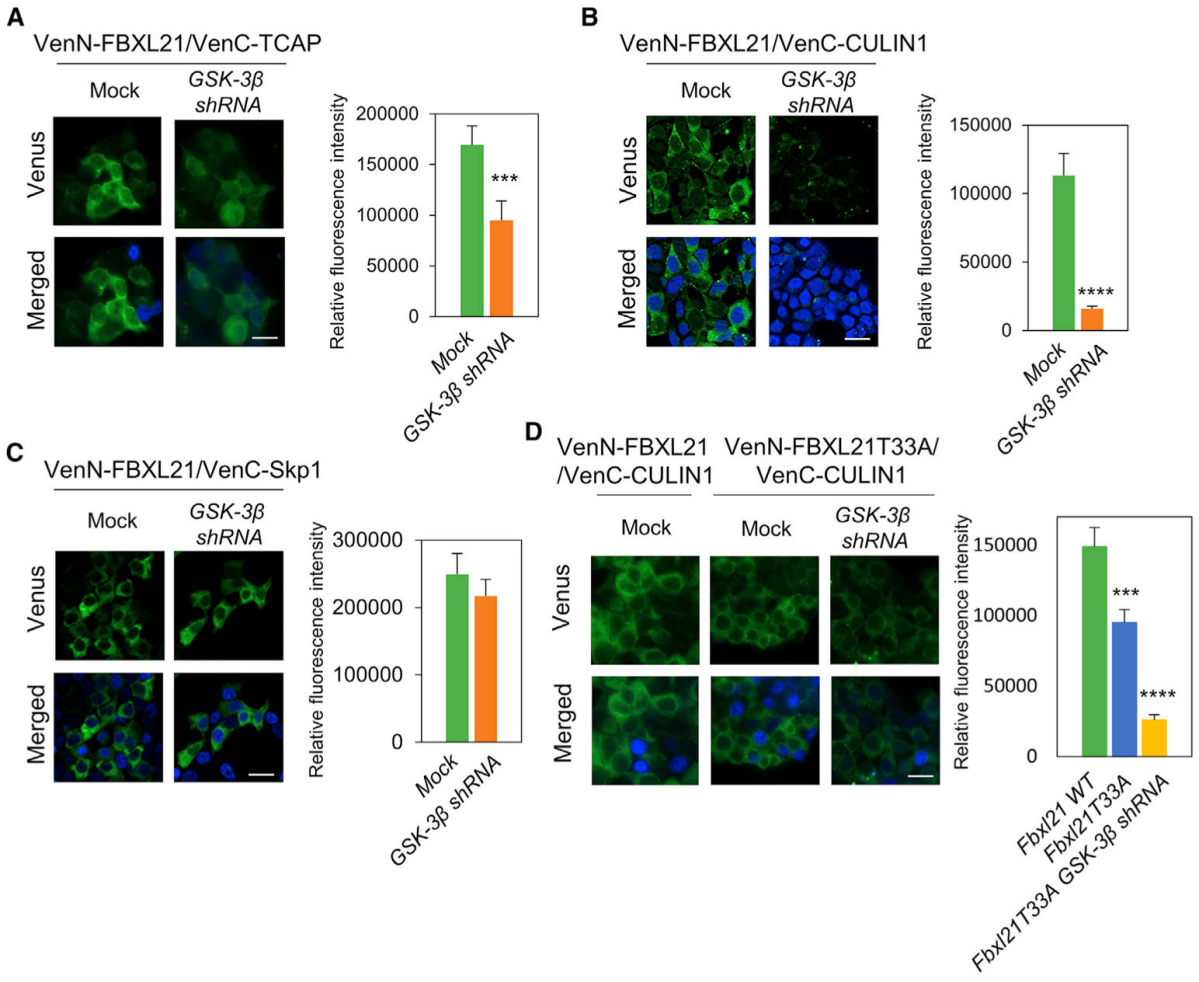
TCAP-FBXL21 and FBXL21-CULLIN1 Complex Formation Is Regulated by GSK-3β Green and blue colors denote Venus and DAPI signals. (A) TCAP and FBXL21 form a complex in the cytoplasm, and shGSK-3β co-transfection inhibits complex formation. Right panel: the bar graph shows mean ± SEM from BiFC signal quantification; n = 3. One-way ANOVA shows that the relative fluorescence intensity is significantly statistically different between mock (scramble shRNA) and GSK-3β shRNA. (***p < 0.001). (B) FBXL21-CULLIN1 complex formation was significantly inhibited by shGSK-3β co-transfection. Right panel: the bar graph shows mean ± SEM of BiFC signal quantification; n = 3. One-way ANOVA shows a significant statistical difference between mock and GSK-3β shRNA (****p < 0.0001). (C) FBXL21-SKP1 complex formation was not affected by shGSK-3β co-transfection. Right panel: the bar graph shows mean ± SEM; n = 3. One-way ANOVA shows that the relative fluorescence intensity is statistically not different. Scale bar, 20 μm. (D) FBXL21T33A showed reduced Cullin1 complex formation compared with FBXL21. Right panel: the bar graph shows mean ± SEM; n = 3. One-way ANOVA shows a significant statistical difference between Fbxl21 WT and Fbxl21T33A (***p < 0.001) and Fbxl21T33A.GSK-3β shRNA (****p < 0.0001). Scale bar, 20 μm. See also Figure S5.

We performed bimolecular fluorescence complementation (BiFC) assay (Kerppola, 2006) to investigate the role of GSK-3β in FBXL21-TCAP and FBXL21-SCF complex formation (Figures 6 and S5). Specifically, VenN-FBXL21 (Venus N terminus fused with Fbxl21) and VenC-TCAP (Venus C terminus fused with TCAP) interaction was measured at the Venus emission wavelength (528 nm). The FBXL21-TCAP complex was observed in the cytoplasm, and GSK-3β shRNA co-transfection significantly inhibited FBXL21-TCAP complex formation (Figure 6A). To examine a causal role of GSK-3β in FBXL21 activity regulation, we used a VenN-Fbxl21 and VenC-Cullin1 BiFC pair, as reported previously (Yoo et al., 2013). FBXL21-CULLIN1 complex formation primarily occurred in the cytoplasm (Figure 6B). GSK-3β knockdown markedly impaired FBXL21-CULLIN1 complex formation, suggesting that GSK-3β mediated FBXL21 phosphorylation is key for FBXL21 activation and SCF complex formation. GSK-3β knockdown did not affect FBXL21-SKP1 complex formation (Figure 6C), suggesting that phosphorylation of FBXL21 is required for the active stage of SCF complex formation (F box-CULLIN1 complex formation) but not the default state of F box protein binding to SKP1 (Reitsma et al., 2017). Finally, the FBXL21 mutant harboring the GSK-3β target site mutation (VenN-FBXL21T33A) showed significantly reduced CULLlN1 complex formation (Figure 6D). GSK-3β shRNA treatment further decreased BiFC signals, suggesting additional GSK-3β phosphorylation sites other than T33.

### Smaller Fiber Size and Reduced Muscle Function in *Psttm* Mutant Mice

**Figure 7.**
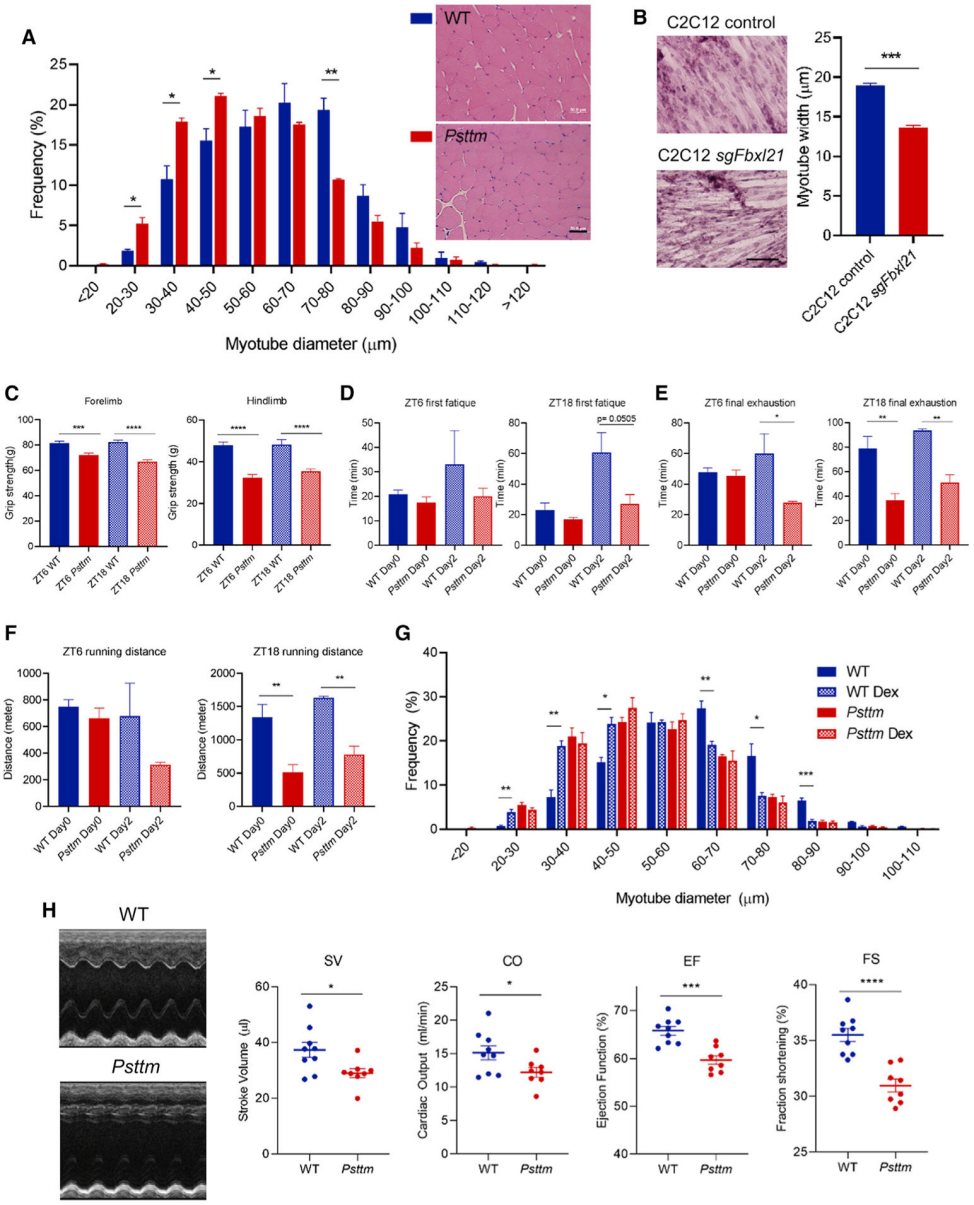
*Psttm* Mice Exhibit Impaired Muscle Function (A) Frequency distribution of myotube diameter in gastrocnemius muscle from WT and *Psttm* mice. A t test shows that the distribution is significantly different between WT and *Psttm* mice at 20–30, 30–40, and 40-50 (*p < 0.05) and 70–80 μm (**p < 0.01). Right: representative H&E staining images from WT and *Psttm* mice. Scale bar, 50 μm. (B) H&E staining images from differentiated control C2C12 cells and CRISPR-Cas9 sgFbxl21 cells. Right panel: quantification of myotube width (***p < 0.001). Scale bar, 100 μm. (C) Grip strength test (n = 10 for WT, n = 11 for *Psttm*). (D–F) Treadmill assays of WT and *Psttm* mice at ZT6 (left panels) and ZT18 (right panels). Data are presented as mean ± SEM (n = 4–5). *p < 0.05, **p < 0.01, ***p < 0.001, ****p < 0.0001. (D) First fatigue measurements. (E) Final exhaustion measurements. (F) Running distance. (G) Frequency distribution of myotube diameter in gastrocnemius muscle from saline- or dexamethasone (Dex)-treated WT and *Psttm* mice (n = 3–4). T test shows that the distribution is significantly different between control and Dex-treated WT mice at 40–50 and 70–80 (*p < 0.05); 20–30, 30–40, and 60–70 (*p < 0.01); and 80–90 (***p < 0.001). (H) Left panels: representative M mode images of WT and *Psttm* heart echocardiographs. Right graphs: cardiac parameter measurements show significant differences between WT (n = 9) and *Psttm* (n = 8) mice for SV, CO, EF, and FS (*p < 0.05, ***p < 0.001, ****p < 0.0001). See also Figures S6 and S7.

To investigate the role of FBXL21 in skeletal muscle *in vivo*, we compared muscle fiber diameter between WT and *Psttm* mice, and the histogram result demonstrated a significant shift to smaller fiber sizes in *Psttm* compared with the WT (Figure 7A). There were differences in fiber diameter between WT and *Psttm* at two circadian time points (ZT6 and ZT18) but no time-of-day effects for either genotype (Figure S6A). Muscle fiber type distribution showed no significant changes between WT and *Psttm* mice (Figure S6B). Next we used CRISPR-Cas9 to delete *Fbxl21* from C2C12 cells to investigate FBXL21 function during myotube differentiation (Figures 7B and S6C). Deletion of *Fbxl21* significantly reduced myotube width, consistent with Figure 7A and supportive of a role of FBXL21 in regulating myotube diameter. On the other hand, in WT mice and control C2C12 cells, muscle fiber diameter did not oscillate as a function of circadian time, at least under the tested conditions (Figures S6D and S6E).

To examine skeletal muscle function, we first subjected WT and *Psttm* mice to a grip test to investigate muscle strength (Figure 7C). Compared with WT littermates, *Psttm* mice showed significantly reduced grip strength for forelimbs and hindlimbs at two circadian time points (ZT6 and ZT18). Next we performed treadmill assays at ZT6 (daytime) and ZT18 (nighttime) to compare their first fatigue, final exhaustion, and running distance. As shown in Figures 7D-7F, we observed significant differences between WT and *Psttm* mice in final exhaustion and running distance, with a trend of difference for first fatigue. The functional differences between the genotypes were generally greater and statistically significant at ZT18, indicating a requisite role of FBXL21 in muscle function during the active phase. For example, WT mice showed a clear circadian pattern of running distance with a strong increase from ZT6 (inactive) to ZT18 (active) (Figures 7F and S6F). Such a circadian pattern was disrupted in *Psttm* mice, resulting in markedly dampened exercise endurance at ZT18. On the other hand, in rotarod tests at both circadian times, we did not observe significant differences between WT and *Psttm* mice (Figure S6G).

Skeletal muscle atrophy is caused by an imbalance of protein synthesis and degradation, and GSK-3β has been shown to function in glucocorticoid-induced muscle atrophy (Bodine and Furlow, 2015; Schakman et al., 2008; Schiaffino et al., 2013; Verhees et al., 2011). We investigated whether FBXL21 may function in dexamethasone (Dex)-induced muscle atrophy (Gilson et al., 2007; Sun et al., 2014). In WT mice, Dex significantly reduced gastrocnemius muscle fiber size. Interestingly however, *Psttm* mice showed resistance to Dex-induced fiber size reduction (Figures 7G and S6H), suggesting a role of the E3 ligase FBXL21 in glucocorticoid-induced muscle atrophy. These results highlight a pivotal physiological function of FBXL21 in skeletal muscle.

Finally, we examined cardiac muscle function. H&E staining, Masson’s trichrome staining, and measurement of left ventricle wall thickness did not reveal significant differences in gross morphology and cardiomyocyte diameter between WT and *Psttm* mice (Figures S7A-S7C). Interestingly, echocardiography showed significant impairments in stroke volume (SV), cardiac output (CO), ejection function (EF), and fraction shortening (FS), indicating an important role of FBXL21 in heart function (Figure 7H). Consistent with the H&E staining results, the echocardiographic dimensions were similar in WT and *Psttm* mice, suggesting that functional impairment precedes structural alteration (Figure S7D). These results support a regulatory role of FBXL21 in cardiac muscle.

## DISCUSSION

Core clock protein turnover, particularly via ubiquitination-mediated proteasomal degradation by E3 ligases, plays critical roles in regulating circadian rhythm (Gallego and Virshup, 2007; He et al., 2005; Hirano et al., 2016; Koh et al., 2006; Liu et al., 2017; Stojkovic et al., 2014). Previously, we reported two homologous E3 ligases, FBXL3 and FBXL21, and demonstrated their antagonistic mechanism to fine-tune CRY turnover equilibrium (Yoo et al., 2013). Here we identified TCAP as a substrate for the cytoplasm-specific E3 ligase activity of FBXL21. We showed circadian oscillation of FBXL21 interaction with TCAP, which is antiphasic to the circadian pattern of TCAP protein accumulation. GSK-3β regulates TCAP degradation by co-phosphorylation of TCAP and FBXL21; the latter was found to be important for FBXL21-CULLIN1 complex formation and activation. *In vivo* studies further revealed a key role of FBXL21 in maintaining muscle fiber size and several skeletal muscle functions, including exercise endurance, grip strength, and atrophy. These experiments illustrate a GSK-3β-FBXL21 functional axis in skeletal muscle. Of note, echocardiography revealed a role of FBXL21 in heart output function, potentially contributing to the observed effects *in vivo*.

Skeletal muscle is increasingly appreciated as a key organ under circadian control. Various aspects of skeletal muscle biology, including gene expression, differentiation, contraction, and metabolism, have been shown to interact with circadian rhythms (Harfmann et al., 2015; Lefta et al., 2011). The current study unveils a molecular pathway at the interface of skeletal muscle biology and circadian rhythms. TCAP is localized at the Z line of the sarcomere and has been implicated in sarcomere assembly. TCAP knockout mice have been found to develop heart failure with biomechanical stress (Knöll et al., 2011), and human mutations in TCAP are associated with a form of autosomal recessive limb-girdle muscular dystrophy (AR LGMD) type 2G and dilated cardiomyopathy (Chamova et al., 2018; Francis et al., 2014; Hershberger et al., 2008; Hirtle-Lewis et al., 2013; Knöll et al., 2002; Markert et al., 2010; Moreira et al., 2000). We show that the circadian E3 ligase FBXL21 governs rhythmic accumulation of TCAP. Disruption of FBXL21 function in skeletal muscle alters TCAP stability and adversely affects muscle structure and function. Several other E3 ligases have been found to interact with TCAP, including MuRF1/2 and MDM2 (Tian et al., 2006; Witt et al., 2005, 2008). MuRF1/2 interaction with TCAP has been found by Y2H, but their direct contribution to TCAP degradation was not validated (Witt et al., 2005). MDM2-mediated TCAP degradation was not dependent on the ring finger domain (Tian et al., 2006), suggesting that MDM2 is not the direct E3 ligase transferring Ub to TCAP. Here we demonstrate that FBXL21 is a requisite E3 ligase for TCAP proteasomal degradation and that the *Psttm* hypomorph mutation disrupts circadian oscillation of TCAP in skeletal muscle. Future studies will investigate the possibility that TCAP oscillation may promote diurnal structural remodeling in the sarcomere and dissect local and systemic (e.g., heart) mechanisms by which TCAP dysregulation in *Psttm* mice contributes to the observed *in vivo* phenotypes, including fiber size and muscle function. Because E3 ligases are known to target many proteins for degradation, investigation of substrates other than TCAP will further define the mechanistic basis of FBXL21 function *in vivo*.

Compared with target substrate identification, upstream regulatory mechanisms governing E3 ligases are often not as well understood. Consistent with the growing evidence highlighting PTMs as key regulatory events for E3 ligases (Fuchs et al., 2002; Gao et al., 2009; Jura et al., 2006; Lin et al., 2009; Wang et al., 2018; Xu et al., 2010), we report that GSK-3β regulates FBXL21-CULLIN1 complex formation as a critical step of E3 ligase activation. TCAP phosphorylation is known to regulate myofibrillogenesis, and PKD has been found to interact with cardiac TCAP and function in the maintenance of transverse tubule organization and intracellular Ca^2+^ transients in myocytes (Candasamy et al., 2014; Mayans et al., 1998). However, a PKD-selective inhibitor did not affect TCAP phosphorylation (Candasamy et al., 2014), suggesting that additional kinases (e.g., GSK-3β) are involved in TCAP phosphorylation. Inhibition of the other kinases tested in our assays did not alter TCAP accumulation. Whether these other kinases serve additional roles in TCAP regulation remains to be investigated.

GSK-3 kinases have been shown to cooperate with the SCF E3 ligase β-TRCP, targeting more than 20 substrates for degradation by β-TRCP (Robertson et al., 2018). GSK-3s regulate circadian rhythms by various mechanisms. In *Drosophila*, the GSK-3 homolog Shaggy (sgg) phosphorylates TIMELESS, which induces nuclear translocation of the PER/TIM dimer (Martinek et al., 2001). In mammals, GSK-3β has been shown to phosphorylate five core clock proteins, including PER2, CRY2, CLOCK, BMAL1 and REV-ERBα (Kaladchibachi et al., 2007; Kurabayashi et al., 2010; Sahar et al., 2010; Spengler et al., 2009; Yin et al., 2006). Double knockin mice expressing constitutively active forms of GSK-3α and GSK-3β showed altered circadian rhythms (Paul et al., 2012). Furthermore, inhibition of GSK-3β can lead to period lengthening by lithium (Li et al., 2012) or shortening (Hirota et al., 2008) by other small-molecule GSK-3β inhibitors. We show here that FBXL21 ubiquitinates TCAP via a GSK-3β target phosphodegron; the FBXL21 E3 ligase activity is itself regulated by GSK-3β phosphorylation, suggesting a co-phosphorylation mechanism. Therefore, although both GSK-3β and FBXL21 regulate the core oscillator and modify rhythmic behavior, our study links them in a functional pathway, revealing circadian regulation of protein turnover by the GSK-3β-FBXL21 axis.

GSK-3 signaling orchestrates broad physiological functions (Beurel et al., 2015). For example, GSK-3β is a negative modulator underlying the anabolic insulin growth factor 1 (IGF-1)/AKT pathway, known to stimulate muscle protein synthesis and inhibit muscle protein degradation (Glass, 2005). A kinase-inactive form of GSK-3β and pharmacological inhibition of GSK-3β induce hypertrophy (Rommel et al., 2001; Vyas et al., 2002) and have been applied as interventions for skeletal muscle atrophy (Verhees et al., 2011). GSK-3β in particular promotes muscle protein degradation in response to glucocorticoids (Schiaffino et al., 2013; Verhees et al., 2011). Although previous studies highlighted a key role of proteasomal degradation in GSK-3β regulated muscle protein turnover, direct regulation of E3 ligase activity by GSK-3 and the molecular mechanism of myofibrillar protein degradation have not been reported. We revealed a direct link of GSK-3β and FBXL21-mediated TCAP degradation and demonstrated defective glucocorticoid-induced muscle atrophy in *Psttm* mice. These results suggest a regulatory function of GSK-3β-FBXL21 that may be targeted to alleviate muscle dystrophy, commonly observed in disease and aging conditions.

In conclusion, we identified a cytoplasmic substrate, TCAP, for the circadian E3 ligase FBXL21, and FBXL21 deficiency led to impaired skeletal muscle function. Mechanistically, we uncovered GSK-3β as a key upstream regulator, phosphorylating TCAP and FBXL21 and promoting SCF complex formation for TCAP degradation. These studies highlight a GSK-3β-FBXL21 axis in circadian regulation of muscle physiology.

## STAR★METHODS

### RESOURCE AVAILABILITY

#### Lead Contact

Further information and requests for resources and reagents should be directed to and will be fulfilled by the Lead Contact (Seung-hee.yoo@uth.tmc.edu).

#### Materials Availability

All unique/stable reagents generated in this study are available upon reasonable request from the Lead Contact. A Materials Transfer Agreement (MTA) may apply.

#### Data and Code Availability

This study did not generate unique datasets or code.

### EXPERIMENTAL MODEL AND SUBJECT DETAILS

#### Animal studies

C57BL/6J (Stock # 000664) mice were purchased from the Jackson Laboratory (Bar Harbor, ME); *Psttm* and littermate WT mice were maintained in house. All mice were housed under LD12:12 unless otherwise noted. All animal studies, using male mice at the age of 10-17 weeks, were approved by UTHealth Center for Laboratory Animal Medicine and Care (CLAMC).

#### Cell culture, differentiation and CRISPR

293T (ATCC: CRL-3216) cells and C2C12 (ATCC: CRL-1772) were cultured in DMEM supplemented with 10% fetal bovine serum (GenDEPOT). For C2C12 differentiation, cells were maintained in DMEM with 10% FBS and penicillin/streptomycin until 90% confluence. Cells were incubated in differentiation media (DMEM containing 2% horse serum and penicillin/streptomycin). Differentiation media were changed daily until cells were fully differentiated (around day 5). Cells were synchronized by Dexamethasone (Dex, 200 nM) for 1 hr, and collected every 4 hr. To generate Fbxl21 CRISPR cell lines, the sense and antisense guide RNAs (Table S1) were designed (https://crispr.dbcls.jp) and cloned into the BsmB1 site of the LentiCRISPR v2. C2C12 cells were transfected and selected for single colonies survived from puromycin selection.

### METHOD DETAILS

#### Yeast 2-hybrid (Y2H) screen

Y2H was performed with the AH109 strain (Panbionet). Bait plasmids express FBXL3 and FBXL21 fused with the GAL4 DNA binding domain. Briefly, Fbxl3 (82-428 aa) and Fbxl21 (86-434 aa) were cloned into EcoRl/BamHI sites of the pGBKT7 vector. Cry2 was cloned into XmaI/SalI digested pGADT7 plasmids to serve as a positive control. The interaction between bait and prey proteins re-constitutes the function of GAL4 and activates reporter gene expression. Positive control yeasts were transformed with the pGBKT7-p53 bait plasmid and the pGADT7-SV40 Tag prey plasmid. pGBKT7-p53 encodes the Gal4 DNA-BD fused with murine p53; pGADT7-T encodes the Gal4 AD fused with SV40 large T-antigen. Cells transformed with the parental bait vector (pGBKT7) and the prey vector (pACT2) were used as negative controls. Transformants were incubated in SD-LW, SD-LWA and SD-LWH media containing 5 mM of 3-AT(3-amino-1,2,3-triazole), a competitive inhibitor of the yeast HIS3 protein (His3p) at 30°C. SD-LW, SD-LWA and SD-LWH indicate synthetic complete media devoid of leucine/tryptophan, leucine/tryptophan/adenine and leucine/tryptophan/histidine respectively. Among > 30 positive interaction hits, we validated interaction of several top candidates by co-IP with FBXLs, with validation of other candidates currently on-going. For this study, we chose to focus on TCAP because it was recently found to be encoded by a clock-controlled gene (Podobed et al., 2014).

#### Cell culture transfection

293T (ATCC CRL-3216) cells and C2C12 (ATCC CRL-1772) were cultured in DMEM supplemented with 10% fetal bovine serum (GenDEPOT). For immunoprecipitations, 2 × 10^6^ cells were plated into 60 mm dishes 1 day before transfection, and iMFectin (GenDEPOT) was used to transfect DNA according to the manufacturer’s protocol. M2 agarose bead (Sigma) was used to pull down flag tagged proteins. TCAP degradation assays were performed by transfecting pCMV10-3XFlag-TCAP with or without Fbxl3 and Fbxl21 expression constructs into 2 × 10^5^ 293T cells in a 12-well plate. Thirty-six hours after transfection 100 μg/ml cycloheximide (CHX) was added and cells collected at the indicated times. The incubation time length was chosen to avoid significant cytotoxicity while still sufficient to distinguish effects. Half-life was determined by using nonlinear, one-phase decay analysis (GraphPad Prism). siRNA for hFbxl21 was purchased from Dharmacon and transfection was performed by using Lipofectamine 2000 (Invitrogen). CHIR99021, Ro3306, NU7441, Sotrastaurin, and IC261 were purchased from Selleckchem.

#### Immunoblotting, immunoprecipitation and immunocytochemistry

Immunoblotting, immunoprecipitation and immunocytochemistry were performed as described previously (Siepka et al., 2007; Yoo et al., 2013). For ectopic expression of Flag-TCAP, Flag-FBXL3 and Flag-FBXL21 we used Flag-HRP antibody (Sigma). For endogenous TCAP expression detection from skeletal muscle and C2C12 samples, we used anti-TCAP antibody (BD). Antibodies against FBXL21 and BMAL1 were generated using guinea pig, rabbit or chicken (Cocalico Biologicals) as described previously (Nohara et al., 2019; Yoo et al., 2013) and serum was affinity purified using the same protein or peptide used to raise the antibody. Commercial vendors of antibodies used include BD (TCAP), Invitrogen (anti-Skp1, anti-Cullin1), Santa Cruz (β-actin, α-tubulin), Abcam (anti-LaminB1), Sigma (anti-Flag-HRP, anti-HA-HRP, M2 bead), GAPDH (Ambion). Nuclear fractionation and ubiquitination assays were performed as described before (Yoo et al., 2013). Muscle fiber typing was performed as described previously (Schroder et al., 2015). We used BA-D5 (Type I), SC-71 (Type IIa) and BF-F3 (Type IIb) from DSHB (Developmental Studies Hybridoma Bank).

#### Liquid Chromatography-Mass Spectrometry Analysis

Affinity-purified proteins were separated by SDS-PAGE. Protein bands were digested in gel with various enzymes including Trypsin, Chymotrypsin or GluC to increase identification coverage. Digested peptides were extracted from gel and separated by Ultimate 3000 nano-LC system (Thermo Scientific). Separated peptides were directly electro-sprayed and injected into Orbitrap Fusion Tribrid system (Thermo Fisher Scientific) in data-dependent mode. Top 50 precursors were fragmented and spectra acquired in CID mode. Obtained spectra were searched and validated by using Proteome Discoverer 1.4 interfaced with Mascot algorism (Mascot 2.4, Matrix Science).

#### *In vitro* kinase assay

Flag-TCAP and Flag-FBXL21 were ectopically expressed and affinity purified from 293T cells, with cells transfected with the Flag-pCMV10 empty vector as a mock control. These proteins and TCAP S161p (GPLRRTLSRSMSpQEAQRG), FBXL21S37p (RGLCSSLRQTHALSpVLLD) peptides (GenScript) were used as substrates for *in vitro* kinase assays by using GSK-3β Kinase Enzyme System with ADP-Glo Kinase Assay (Promega) or Z’-Lyte Kinase Assay (Invitrogen).

#### TCAP and Fbxl21 mutants

Site-directed mutagenesis of Flag-TCAP, Flag-Fbxl21, VenusN-Fbxl21, and VenusN-TCAP to construct lysine or serine revertant clones and FBXLs chimeric construct were performed by using primers listed in Table S1.

#### Bimolecular Fluorescence Complementation (BiFC) Assay

BiFC experiments were performed as described previously (Yoo et al., 2013). PCR amplified TCAP cDNA was cloned into KpnI and BamHI sites of VenC. Briefly, VenN-TCAP or VenN-Fbxls and VenC-Skp1 or VenC-Cullin were transfect to 3x10^5^ 293T cells using iMFectin (GenDEPOT). GSK-3β shRNAs (pLKO.1-GSK-3β-1, Addgene #32496; pLKO.1-GSK-3β-2, Addgene #32497) are co-transfected in indicated experiments. Media were changed after overnight incubation at 37°C, 5% CO2. Plates were washed with PBS once 2 days after transfection and fixed with 4% paraformaldehyde in PBS for 15 min. Samples were stained with DAPI (1 ug/ml), washed twice, immersed with PBS and sealed with top-seal A membrane (Perkin-Elmer). Fluorescence images were acquired with Zeiss Imager.M2m equipped with ApoTome.2, Axiocam 506 mono and AxioCam MRc. Images were captured using Zen2 Software. Seven locations in each slide were selected. Images were taken in the channel sequence of FITC (Ex 493, Em 528) and DAPI (Ex 390, Em 435) filter sets. For quantification of BiFC results, original image files were imported to NIH ImageJ (Schneider et al., 2012) for quantification. Briefly, cytoplasm were recognized based on FITC fluorescence (regions with no fluorescence considered as background) and corrected total cell fluorescence (CTCF) was calculated by integrated density. About 100 cells were identified and measured in each image, and the average values of cells in one image were obtained for further normalization and statistical analysis. Seven images from each slide were averaged and the experiments were repeated three times.

#### Real-time qPCR

Total RNA was extracted from cells or frozen tissue by using TRizol (Invitrogen). Two microgram of total RNA were used for cDNA synthesis (GenDEPOT). Gene expression was analyzed by using Stratagene Mx3000p (Agilent).

#### Muscle fiber and myotube measurements

WT C57BL/6J (n = 3) and homozygous *Psttm* (n = 3) littermate mice at 12-13 weeks of age were sacrificed. Gastrocnemius muscles were isolated in 10% Formaldehyde and embedded in paraffin. Cross sections (10 μM) of the gastrocnemius muscle were stained with Hematoxylin and Eosin and images were acquired at 100x magnification using a high-resolution camera Olympus DP71 (Olympus) attached to a BX60 microscope (Olympus). Five hundred myotube fibers were analyzed from each animal and average muscle fiber diameters were quantified using ImageJ (NIH). For Dex-induced muscle atrophy experiment (n = 3), WT and *Psttm* littermate mice were injected s.c. with either saline (0.9% NaCl) or Dex (5 mg/kg) for 7 days, and muscle fiber size measurements were conducted as above. C2C12 control and sgRNA(Fbxl21 CRISPR) cell lines were cultured and differentiated as indicated above. Fully differentiated cells were fixed with 4% Paraformaldehyde, stained with Hematoxylin and Eosin and images were acquired and analyzed as above.

#### Treadmill exercise, grip strength, and Rotarod tests

Treadmill exercise test was performed largely as previously described (Nohara et al., 2019) with minor modifications. WT (n = 5) and *Psttm* (n = 4) mice at ~16 weeks of age were acclimated to the Exer-3R treadmill (Columbus Equipment) for 3 times over 4 days. After a week of recovery, the mice were tested in the treadmill with an initial speed of 7 m/ min. The speed was gradually increased at the rate of 2 m/min every 5 minutes. The assay was done twice at both ZT6 and ZT18 with two weeks of recovery between the different time points. Three measurements, including first fatigue (the first 15 s that mice stay at the electric stimulation area), final exhaustion (the total running time of the mice) and running distance, were taken for each assay.

For grip test, WT (n = 11) and *Psttm* (n = 12) adult mice were tested by using BIO-GS3 Grip Test instrument (Bioseb). Each test were performed twice at ZT6 and ZT18. At each time point, three measurements were taken for both forelimb and hindlimb.

For Rotarod test, WT (n = 11) and *Psttm* (n = 12) adult mice were tested by using Touchscreen Rotarod (Panlab/ Harvard Apparatus). The mice were first acclimated to the stationary rod for 1 min at a base speed of 4 rpm for 5 mins. After the acclimation, the mice were tested with the increasing speed of 4 - 40 rpm for 5 mins. The latency to fall for each mouse was measured for two days at both ZT6 and ZT18.

#### Echocardiography and heart histology

Heart function in WT and *Psttm* littermate mice (age: 10 weeks) was evaluated with echocardiomyography. M-Mode echocardiographic images were recorded using Vevo 770 echocardiography equipped with a 40-MHz RMV704 ultrasonic probe (Visual Sonics, Toronto, Canada). M-mode images were then captured in the long axes. Each image loop provided 15 to 20 heart cycles; M-mode data were averaged from at least 3 cycles per loop. The cardiac parameter including left ventricle ejection function (EF), fraction shortening (FS), cardiac output (CO) and stroke volume (SV), heart dimensions such as intraventricular septum (IVS), left ventricular internal dimension (LVID) and left ventricle posterior wall (LVPW) were measured according to the American Society of Echocardiography’s leading edge technique.

For heart histology, WT C57BL/6J and *Psttm* (n = 5) littermate mice, age 16-17 weeks of age, were sacrificed. Hearts were isolated, fixed with 10% Formaldehyde and embedded in paraffin. Heart coronal sections were stained with Hematoxylin and Eosin and Masson Trichome. Images were captured with Aperio LV1 (Leica Biosystems) and quantified using LV1 console (Leica Biosystems).

### QUANTIFICATION AND STATISTICAL ANALYSIS

Results are presented as mean ± SEM unless otherwise stated. Data were analyzed using Student’s t test, one-way ANOVA followed by post hoc analysis using Dunnett’s multiple comparison test or two-way ANOVA followed by post hoc analysis using Bonferroni test as offered by GraphPad Prism. A value of p < 0.05 was considered statistically significant.

## Supplementary Material

1

## Figures and Tables

**Table:** KEY RESOURCES TABLE

REAGENT or RESOURCE	SOURCE	IDENTIFIER
Antibodies		
Rabbit polyclonal anti-Skp1	Cell Signaling Technology	Cat# 2156; RRID:AB_2270271
Rabbit polyclonal anti-CUL1	Cell Signaling Technology	Cat# 4995; RRID:AB_2261133
Mouse monoclonal anti-β-Actin (C4)	Santa Cruz Biotechnology	Cat# sc-47778; RRID:AB_2714189
Mouse monoclonal anti-α Tubulin (B-7)	Santa Cruz Biotechnology	Cat# sc-5286; RRID:AB_628411
Rabbit polyclonal anti-Lamin B1	abcam	Cat# ab16048; RRID:AB_443298
Mouse monoclonal anti-FLAG M2-HRP	Sigma	Cat# A8592; RRID:AB_439702
Rat monoclonal anti-HA-Peroxidase (3F10)	Sigma	Cat# 12013819001; RRID:AB_390917
Mouse monoclonal anti-GAPDH Monoclonal Antibody (6C5)	Thermofisher	Cat# AM4300; RRID:AB_2536381
Mouse monoclonal anti-Telethonin (clone 53)	BD	Cat# 612328; RRID:AB_399643
Rabbit polyclonal anti-GSK-3β (D5C5Z)	Cell Signaling Technology	Cat# 12456; RRID:AB_2636978
Chicken polyclonal anti-BMAL1	Nohara et al., 2019	N/A
Guinea pig polyclonal anti-FBXL3	Yoo et al., 2013	N/A
Rabbit polyclonal anti-FBXL21	Yoo et al., 2013	N/A
Mouse monoclonal anti-myosin heavy chain Type I	DSHB	BA-D5; RRID:AB_2235587
Mouse monoclonal anti-myosin heavy chain Type IIa	DSHB	SC-71; RRID:AB_2147165
Mouse monoclonal anti-myosin heavy chain Type IIb	DSHB	BF-F3; RRID:AB_2266724
Bacterial and Virus Strains		
Yeast strain AH109	Panbionet	N/A
Yeast strain PBN204	Panbionet	N/A
Bacteria strain Stbl3	ThermoFisher	Cat# C737303
Biological Samples		
C57BL/6J, skeletal muscle tissue from mouse	JAX	Cat# 000664
WT littermates of *Psttm* homizygouse mice, skeletal muscle tissue from mouse	In house	N/A
*Psttm* homozygous mice, skeletal muscle tissue from mouse	In house	N/A
Chemicals, Peptides, and Recombinant Proteins		
CHIR-99021	Selleckchem	Cat# S2924
Ro3306	Selleckchem	Cat# S7747
NU7441	Selleckchem	Cat# S2638
Sotrastaurin	Selleckchem	Cat# S2791
IC261	Selleckchem	Cat# S8237
TCAP S161p: (GPLRRTLSRSMSpQEAQRG)	GenScript	N/A
FBXL21S37p (RGLCSSLRQTHALSpVLLD)	GenScript	N/A
Critical Commercial Assays		
GSK-3β Kinase Enzyme System	Promega	Cat# V1991
ADP-Glo Kinase Assay	Promega	Cat# V6930
Z-lyte Kinase Assay	Invitrogen	Cat# PV3324
Experimental Models: Cell Lines		
Human: 293T cells	ATCC	CRL-3216; RRID:CVCL_0063
Mouse: C2C12	ATCC	CRL-1772; RRID:CVCL_0188
Experimental Models: Organisms/Strains		
Mouse: C57BL/6J	The Jackson Laboratory	JAX 000664; RRID:IMSR_JAX:000664
Mouse: *Psttm* mutant	In house, also at the Jackson Laboratory	JAX 016171; RRID:IMSR_JAX:016171
Oligonucleotides		
hFbxl21-F: GGATTAGATGGTCGATATGCC	This paper	N/A
hFbxl21-R: CAGAATCCAAGTTCAATATTACACAT	This paper	N/A
hGAPDH-F: ACAGTCAGCCGCATCTTCTT	This paper	N/A
hGAPDH-R: ACGACC AAATCCGTTGACTC	This paper	N/A
mGAPDH-F: CAAGGAGTAAGA AACCCTGGACC	This paper	N/A
mGAPDH-R: CGAGTTGGGATAG GGCCTCT	This paper	N/A
mTCAP-F: GATGCGCCTGGGTATCCTC	This paper	N/A
mTCAP-R: GATCGAGACAGGGTACGGC	This paper	N/A
Recombinant DNA		
pCMV10-3XFlag-TCAP	This paper	N/A
VenusN-TCAP	This paper	N/A
VenusN-Fbxl21	This paper	N/A
LentiCRISPR v2	Addgene	Cat#52961; RRID:Addgene_80089
pLKO.1-GSK-3β-1	Addgene	Cat# 32496
pLKO.1-GSK-3β-2	Addgene	Cat# 32497
Software and Algorithms		
ImageJ	Schneider et al., 2012	https://imagej.nih.gov/ij/
GraphPad Prism 7	GraphPad Software, Inc	https://www.graphpad.com/

## Highlights

- FBXL21 governs circadian degradation of the sarcomere protein TCAP
- GSK-3β functions upstream of FBXL21 to regulate SCF complex formation
- A role of GSK-3β in circadian rhythm via co-phosphorylation of FBXL21 and TCAP
- Hypomorphic Fbxl21 mutant mice have impaired muscle function
