# Supplementary material for: The GSK-3β-FBXL21 Axis Contributes to Circadian TCAP Degradation and Skeletal Muscle Function
Source: Cell Rep. Author manuscript; Available in PMC 2021 Jul 23. (PMC8299398; doi:10.1016/j.celrep.2020.108140)
Supplement: 1 [file NIHMS1629510-supplement-1.pdf]

Cell Reports, Volume *32*

Supplemental Information

The GSK-3β-FBXL21 Axis Contributes to Circadian TCAP Degradation and Skeletal Muscle Function

Marvin Wirianto, Jiah Yang, Eunju Kim, Song Gao, Keshav Raj Paudel, Jong Min Choi, Jeehwan Choe, Gabrielle F. Gloston, Precious Ademoji, Randika Parakramaweera, Jianping Jin, Karyn A. Esser, Sung Yun Jung, Yong-Jian Geng, Hyun Kyoung Lee, Zheng Chen, and Seung-Hee Yoo

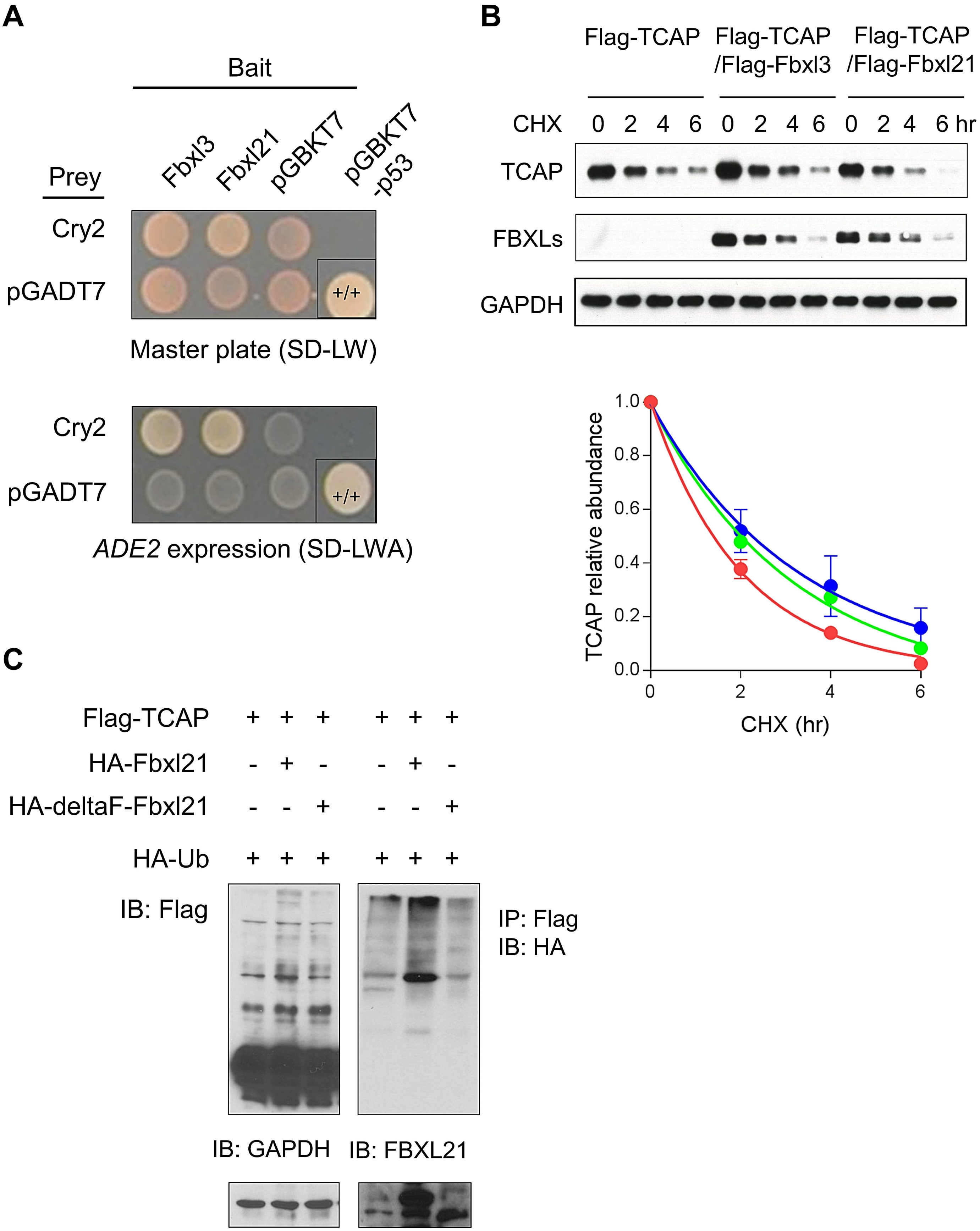
A
Bait
Prey
Fbxl3
Fbxl21
pGBKT7
pGBKT7 -p53
Cry2
pGADT7
+/+
Master plate (SD-LW)
Cry2
pGADT7
+/+
ADE2 expression (SD-LWA)
B
Flag-TCAP
Flag-TCAP /Flag-Fbxl3
Flag-TCAP /Flag-Fbxl21
CHX 0 2 4 6 0 2 4 6 0 2 4 6 hr
TCAP
FBXLs
GAPDH
TCAP relative abundance
1.0
0.8
0.6
0.4
0.2
0.0
0
2
4
6
CHX (hr)
C
Flag-TCAP + + + + + +
HA-Fbxl21 - + - - + -
HA-deltaF-Fbxl21 - - + - - +
HA-Ub + + + + + +
IB: Flag
IP: Flag
IB: HA
IB: GAPDH
IB: FBXL21

Figure S1, related to Figures 1 and 2. (**A**) Y2H test showing FBXL3 and FBXL21 interact with CRY2. (**B**) Differential effects of FBXL3 and FBXL21 on TCAP stability in C2C12 cells. C2C12 cells were co-transfected with the indicated constructs. Thirty two hours after transfection, cells were treated with 100 μg/ml cycloheximide and incubated for the indicated time before harvest. Immunoblotting was performed to detect TCAP and FBXL levels using anti-Flag antibody. Right: quantification of the effect of FBXL3, and FBXL21 on TCAP stability. Error bars represent ± SEM (n=3). Half-life was determined by using nonlinear, one-phase decay analysis (TCAP: 2.3 hrs, TCAP/FBXL3: 1.9 hrs, TCAP/FBXL21: 1.3 hrs; comparison of fits: different curve fit for each dataset, $p<0.0001$). (**C**) FBXL21-mediated TCAP ubiquitination is dependent on F-box. 293T cells were transfected with the indicated constructs. Cells were treated with MG132 (10μg/ml) for 6 hrs before harvest. Cell lysates were immunoprecipitated with anti-Flag antibody and immunoblotting was performed by the indicated antibodies.

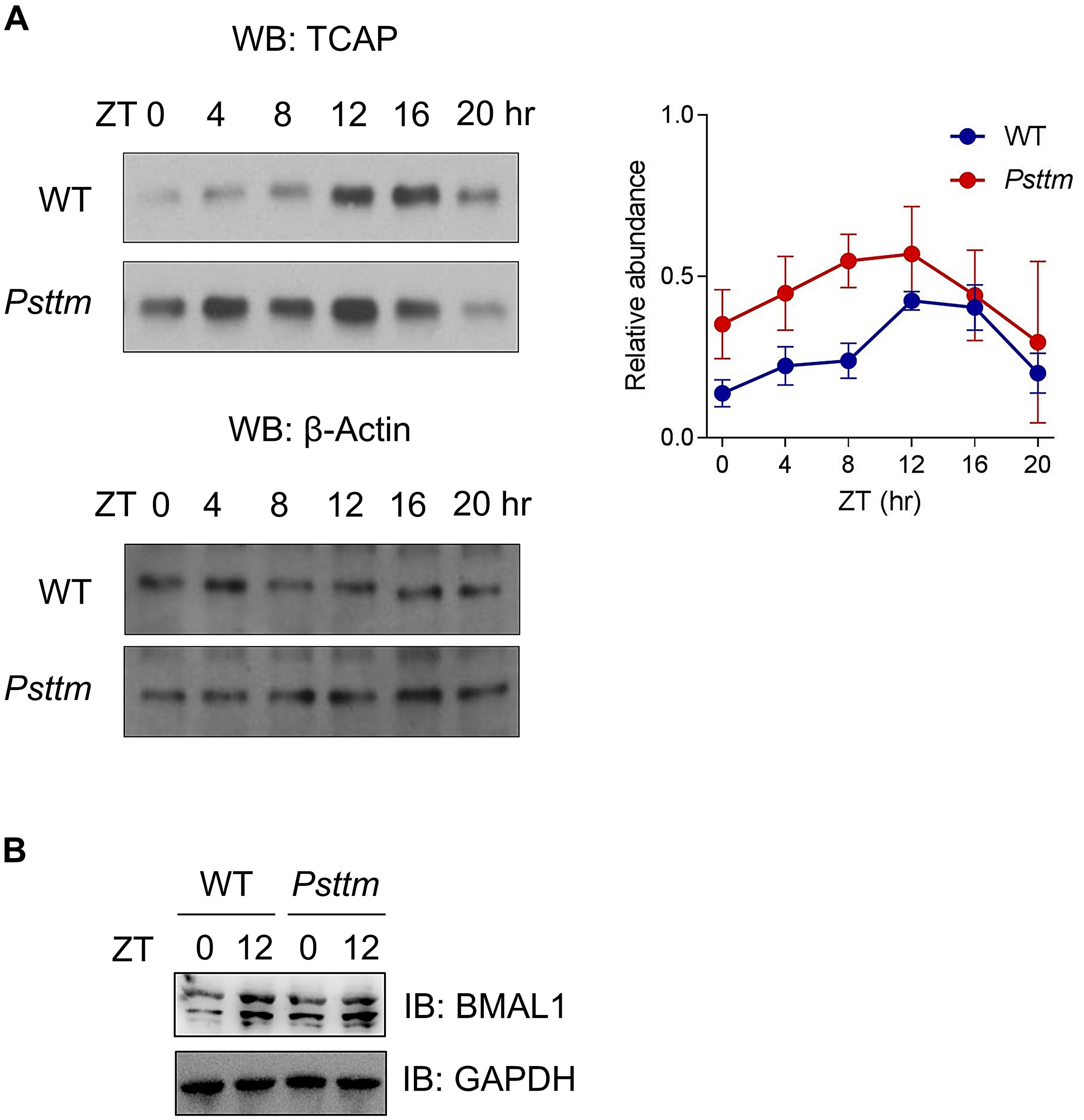

Figure S2, related to Figure 3. (**A**) TCAP oscillation in the heart from WT and *Psttm* mutant mice. Immunoblotting was performed using total protein extracts with anti-TCAP antibody. Representative blots from three independent experiments are shown, and quantification is shown to the right panel (Error bars represent ± SEM). Blue and red circles represent WT and *Psttm* mice respectively. One-way ANOVA with Tukey's post hoc analysis shows significant statistical differences of TCAP amount between time points in WT (*, $p<0.05$) but not in *Psttm*. (**B**) BMAL1 immunoblotting using skeletal muscle tissues collected from WT and *Psttm* mutant mice.

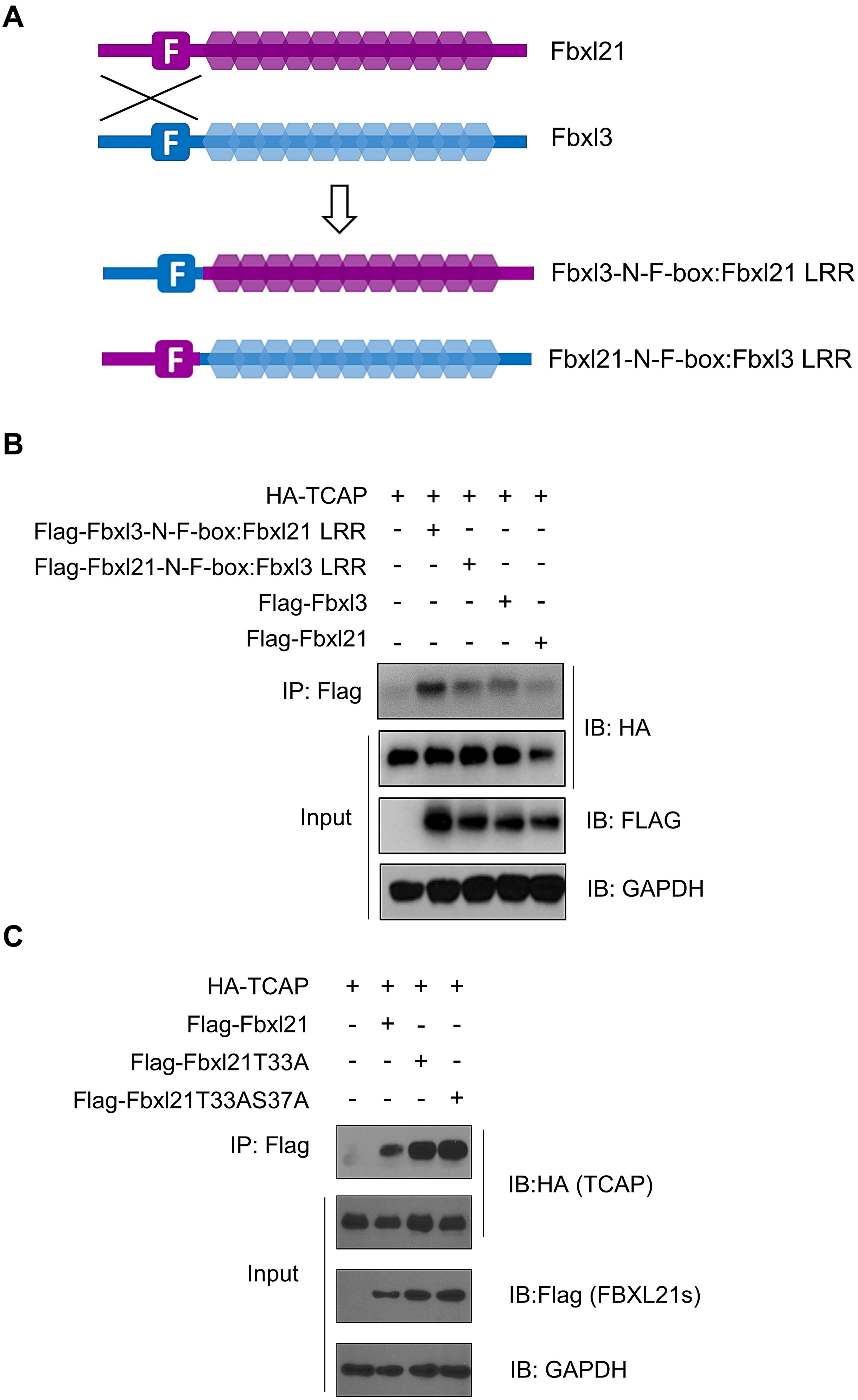

A
Fbxl21
Fbxl3
Fbxl3-N-F-box:Fbxl21 LRR
Fbxl21-N-F-box:Fbxl3 LRR
B
HA-TCAP + + + + +
Flag-Fbxl3-N-F-box:Fbxl21 LRR - + - - -
Flag-Fbxl21-N-F-box:Fbxl3 LRR - - + - -
Flag-Fbxl3 - - - + -
Flag-Fbxl21 - - - - +
IP: Flag
IB: HA
Input
IB: FLAG
IB: GAPDH
C
HA-TCAP + + + +
Flag-Fbxl21 - + - -
Flag-Fbxl21T33A - - + -
Flag-Fbxl21T33AS37A - - - +
IP: Flag
IB:HA (TCAP)
Input
IB:Flag (FBXL21s)
IB: GAPDH

Figure S3, related to Figure 5. The FBXL21 N-terminal domain contains E3 ligase activity for TCAP. (**A**) FBXL3 and FBXL21 contain the F-box domain (boxes) and 11 leucine-rich repeats (LRR) (hexagons). The Fbxl3-N-F-box:Fbxl21 LRR chimeric construct contains Fbxl3 N-terminal domain and Fbxl21 LRR, and the Fbxl21-N-F-box:Fbxl3 LRR chimeric construct contains Fbxl21 N-terminal domain with Fbxl3 LRR. (**B**) Interaction of TCAP with FBXL chimeric proteins. 293T cells were co-transfected with the indicated constructs and immunoprecipitation was performed using anti-Flag antibody (M2). Co-immunoprecipitated proteins were analyzed by immunoblotting with anti-HA antibody. (**C**) Interaction of TCAP with FBX21T33A and FBXL21T33AS37A. 293T cells were co-transfected with the indicated constructs and immunoprecipitation was performed using anti-Flag antibody (M2). Co-immunoprecipitated proteins were analyzed by immunoblotting with anti-HA antibody.

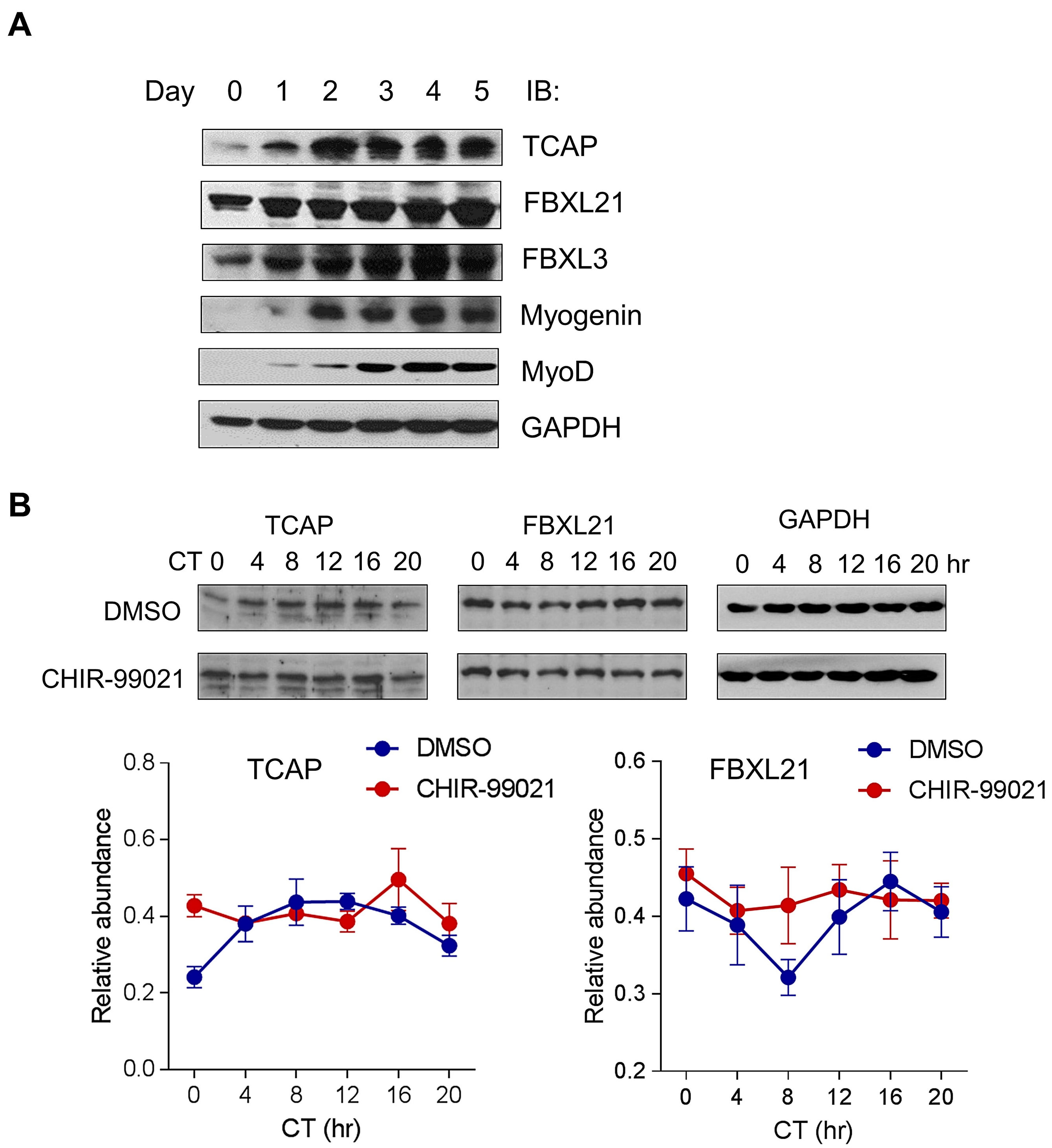

Figure S4, related to Figure 5. TCAP and FBXL21 expression in differentiated C2C12 Cells. (**A**) TCAP expression during C2C12 cell differentiation. Confluent C2C12 cells were treated with 2% horse serum supplemented DMEM for the indicated days. Harvested cells were lysed for immunoblotting with the indicated antibodies. (**B**) After 3 days of differentiation, dexamethasone (200 nM) was used to synchronize cells (1 hr), and cells were harvested at the indicated circadian times (CT). Treatment of the GSK-3β inhibitor CHIR-99021 disrupted circadian oscillation of TCAP and FBXL21 in differentiated C2C12 cells. Representative blots from three independent experiments are shown, and quantification is shown in the right panel (Error bars represent ± SEM). Blue and red circles represent DMSO and CHIR-99021 treatments respectively.

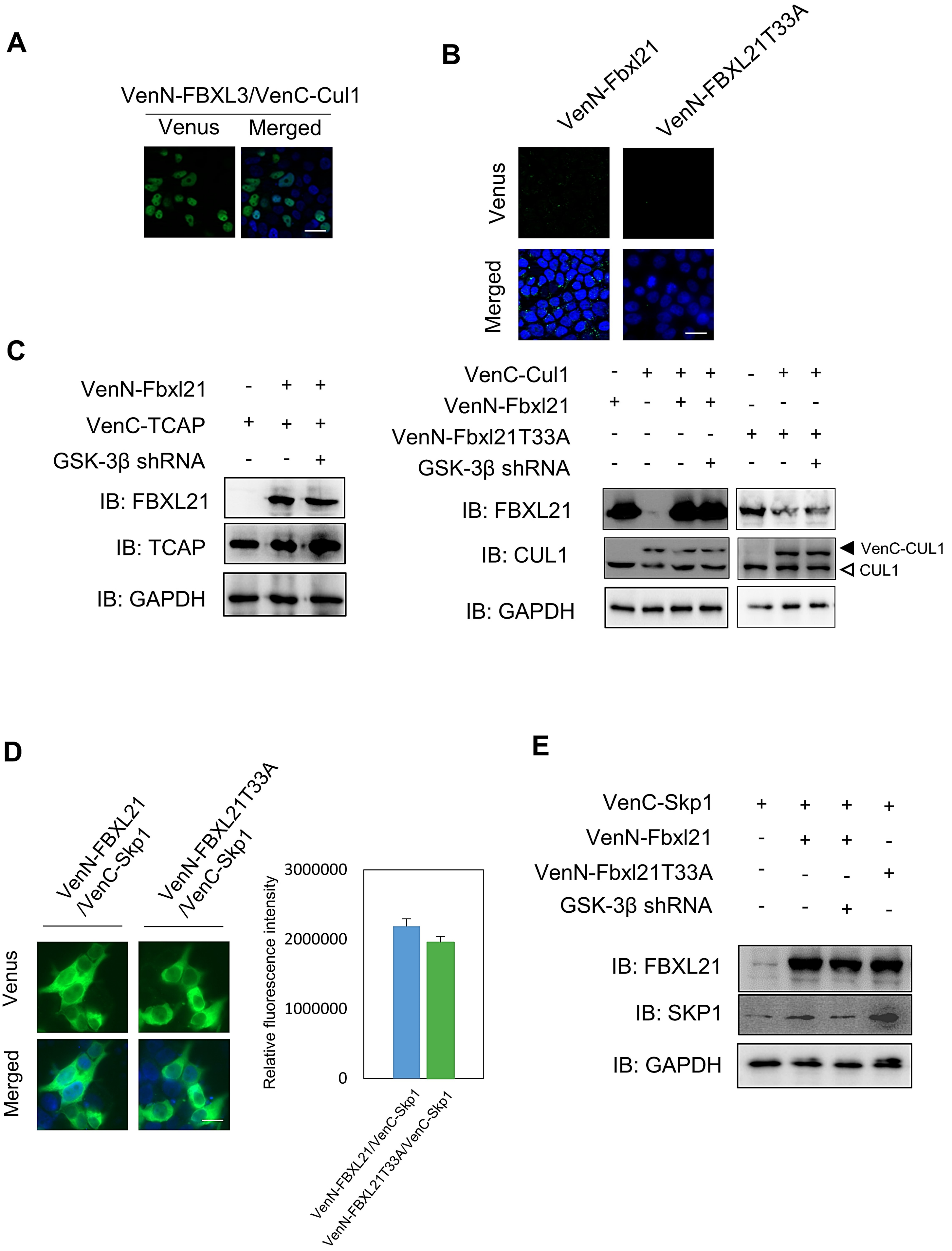
A
VenN-FBXL3/VenC-Cul1
Venus
Merged
B
VenN-Fbxl21
VenN-FBXL21T33A
Venus
Merged
C
VenN-Fbxl21 - + +
VenC-TCAP + + +
GSK-3β shRNA - - +
IB: FBXL21
IB: TCAP
IB: GAPDH
VenC-Cul1 - + + + - + +
VenN-Fbxl21 + - + + - - -
VenN-Fbxl21T33A - - - - + + +
GSK-3β shRNA - - - + - - +
IB: FBXL21
IB: CUL1
VenC-CUL1
CUL1
IB: GAPDH
D
VenN-FBXL21 /VenC-Skp1
VenN-FBXL21T33A /VenC-Skp1
Venus
Merged
Relative fluorescence intensity
3000000
2000000
1000000
0
VenN-FBXL21/VenC-Skp1
VenN-FBXL21T33A/VenC-Skp1
E
VenC-Skp1 + + + +
VenN-Fbxl21 - + + -
VenN-Fbxl21T33A - - - +
GSK-3β shRNA - - + -
IB: FBXL21
IB: SKP1
IB: GAPDH

Figure S5, related to Figure 6. BiFC construct expression validation. (**A**) VenN-Fbxl3 and VenC-Cul1 BiFC). Scale bar, 40 μm. (**B**) VenN-Fbxl21, VenN-Fbxl21T33A, VenN-Fbxl21T33AS37A, and VenC-Cul1 expression validation. 293T cells were co-transfected with the indicated constructs. Thirty two hours after transfection, cells were harvested. Immunoblotting was performed to detect CUL1 and FBXL21. Scale bar, 40 μm. (**C**) VenC-TCAP expression. 293T cells were co-transfected with the indicated constructs. Thirty two hours after transfection, cells were harvested. Immunoblotting was performed to detect VenC-TCAP, FBXL21 and SKP1. (**D**) SKP1 complex formation was not affected by the FBXL21T33A mutation. Right panel: bar graph show the mean ±SEM of quantification of BiFC signals from three replicate experiments. Green: Venus, Blue: DAPI. One-way ANOVA shows the relative fluorescence intensity is statistically not significant. Scale bar, 20 μm. (**E**) VenN-Fbxl21 and VenN-Fbxl21T33A expression. 293T cells were co-transfected with the indicated constructs. Thirty two hours after transfection, cells were harvested. Immunoblotting was performed to detect FBXL21.

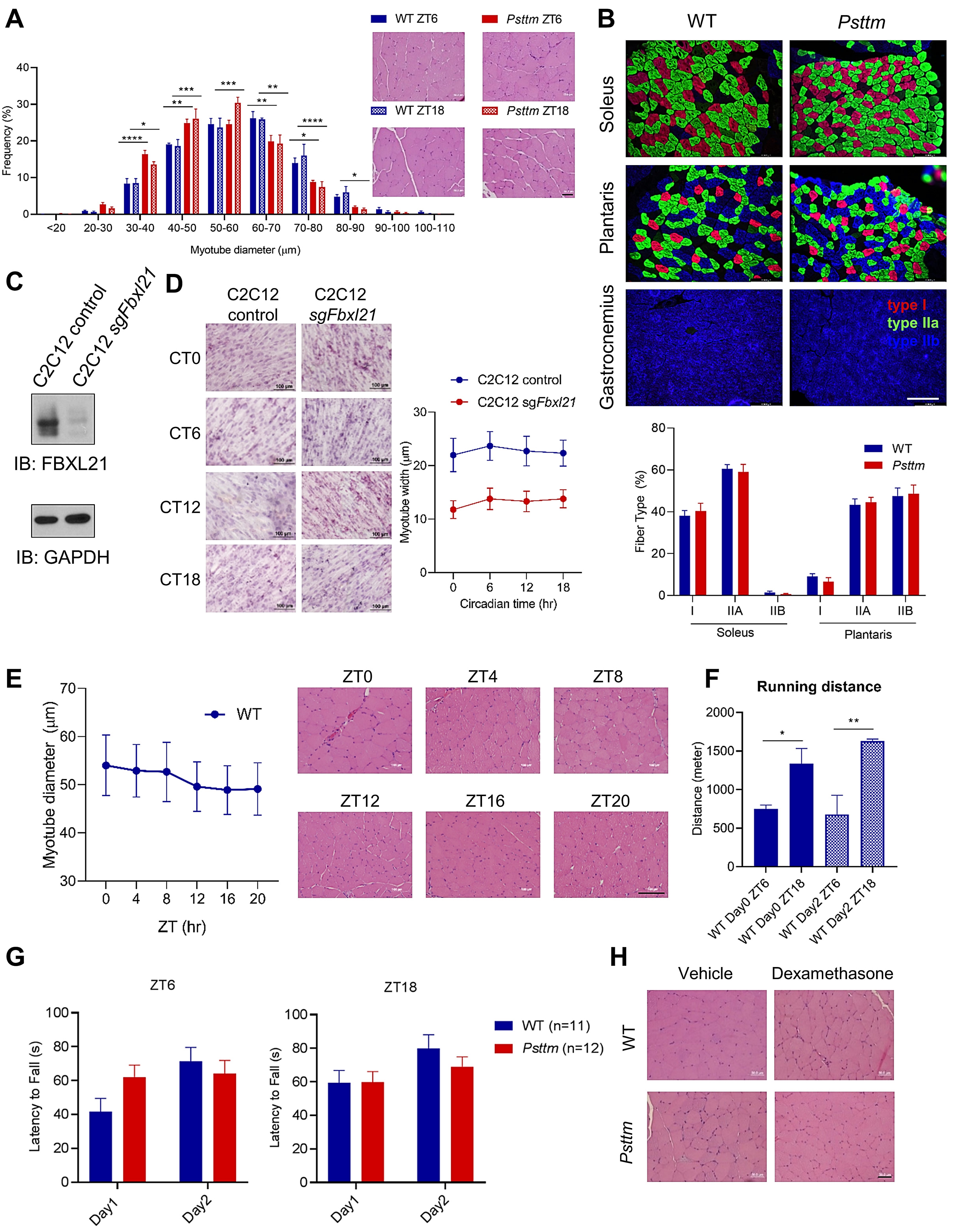

A
WT ZT6
Psttm ZT6
WT ZT18
Psttm ZT18
Frequency (%)
Myotube diameter (μm)
<20
20-30
30-40
40-50
50-60
60-70
70-80
80-90
90-100
100-110
B
WT
Psttm
Soleus
Plantaris
Gastrocnemius
type I
type IIa
type IIb
Fiber Type (%)
I
IIA
IIB
Soleus
Plantaris
C
C2C12 control
C2C12 sgFbxl21
IB: FBXL21
IB: GAPDH
D
C2C12 control
C2C12 sgFbxl21
CT0
CT6
CT12
CT18
Myotube width (μm)
Circadian time (hr)
E
WT
Myotube diameter (μm)
ZT (hr)
ZT0
ZT4
ZT8
ZT12
ZT16
ZT20
F
Running distance
Distance (meter)
WT Day0 ZT6
WT Day0 ZT18
WT Day2 ZT6
WT Day2 ZT18
G
ZT6
ZT18
Latency to Fall (s)
Day1
Day2
WT (n=11)
Psttm (n=12)
H
Vehicle
Dexamethasone
WT
Psttm

Figure S6, related to Figure 7. (**A**) Gastrocnemius muscle fiber diameter distribution in WT and *Psttm* mice. Muscle samples were collected at ZT6 and ZT18. Two-way ANOVA with Tukey's post hoc analysis shows the distribution is significantly different between WT and *Psttm* at different myotube diameter ranges (*, $p<0.05$; **, $p<0.01$; ***, $p<0.001$; ****, $p<0.0001$). Scale bar, 50 μm. (**B**) Representative images from soleus, plantaris, and gastrocnemius muscles from WT and *Psttm* mice. Staining was performed using antibodies against fiber type-specific myosin heavy chain to distinguish type I (red), type IIa (green) and type IIb (blue). Right panel: quantification of fiber type composition. Two-way ANOVA with Sidak's multiple comparison test shows that the fiber type percentages are statistically not different between WT and *Psttm.* Error bars represent ± SEM (n=3). Scale bar, 138 μm (40x). (**C**) FBXL21 expression from control and *Fbxl21*-deleted CRISPR cells (*sgFbxl21*). (**D**) H&E staining images of control C2C12 and *sgFbxl21* cells in different circadian time points. Scale bar, 100 μm. Right panel: quantification of myotube width from control and *sgFbxl21* C2C12 cells. Error bars represent ±SEM (n=3). Two-way ANOVA with Sidak's multiple comparison test shows the distribution is significantly different between control and *sgFbxl21* C2C12 cells at CT0, CT6, and CT12 (*, $p<0.05$). (**E**) Myotube diameter of WT mice collected at different time points. Error bars represent ±SEM (n=4). One-way ANOVA shows that the myotube diameters between different time points are statistically not different. Scale bar, 100 μm. (**F**) Diurnal variation of running distance from WT mice (Data reported from Figure 7F). (**G**) Rotarod test of WT and *Psttm* mice at ZT6 and ZT18 (WT: n=11, *Psttm*: n=12). T-test shows that latency to fall are statistically not different between WT and *Psttm.* (**H**) Representing H&E staining images from Dexamethasone treated WT and *Psttm* mice (n=3-4). Scale bar, 50 μm.

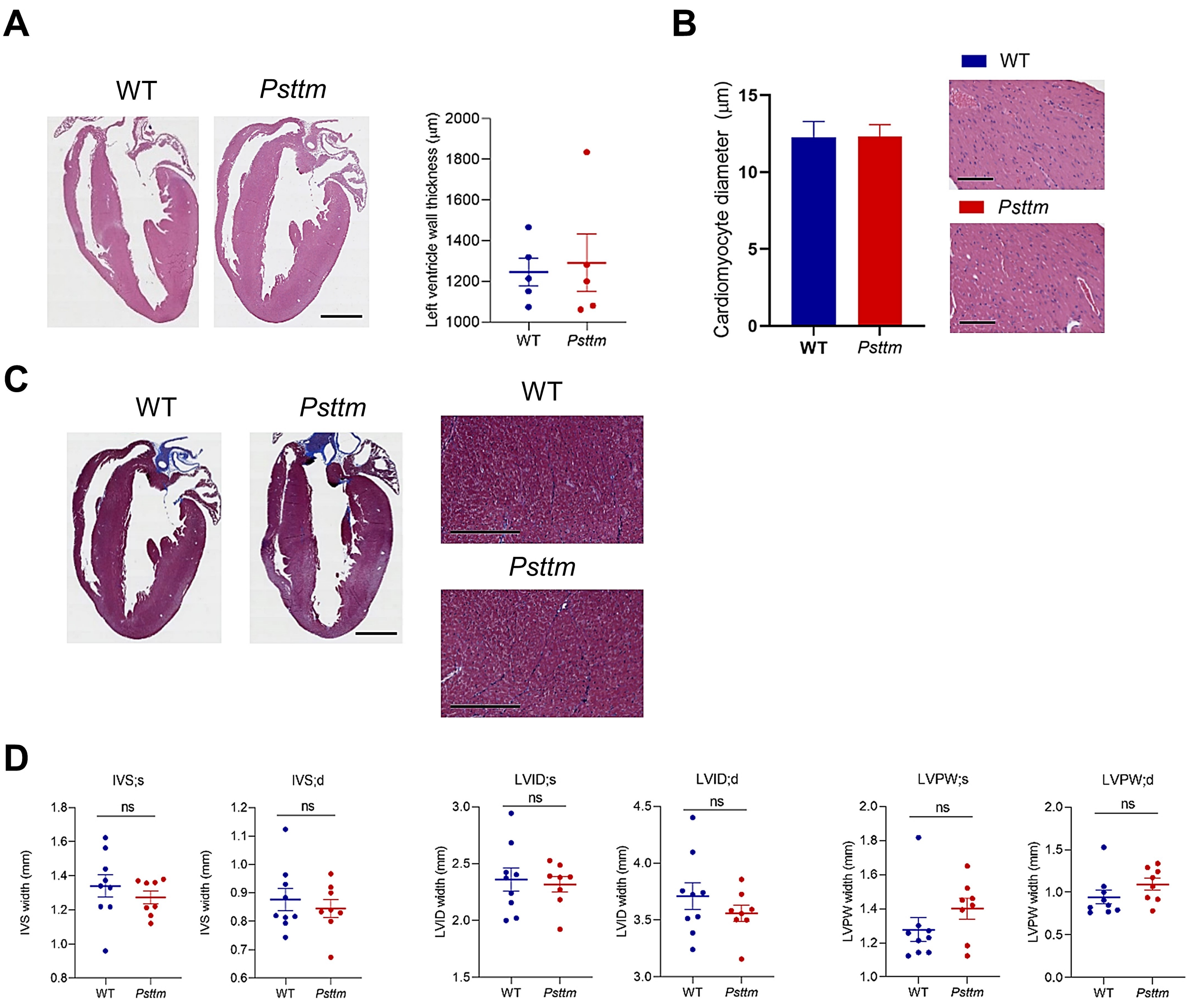

Figure S7, related to Figure 7. (**A**) Representative H&E staining images of heart tissues from WT and *Psttm* mice. Right panel: quantification of left ventricle wall thickness. T-test shows that the left ventricle wall thickness is statistically not different between WT and *Psttm* (n=5). Scale bar, 1.5 mm. (**B**) Quantification of cardiomyocyte diameter (n=5); bar graph show the mean ± SEM. T-test shows that the cardiomyocyte diameter is statistically not different between WT and *Psttm* (n=5). Scale bar, 100 µm. (**C**) Representative images of the Masson's trichrome staining from WT and *Psttm* mice. Left: Scale bar, 1.5 mm. Right: Scale bar, 200 µm. (**D**) Representative M-mode of echocardiography *Psttm* and WT. IVS: left ventricle Intraventricular septum, LVID: Left ventricle Internal Diameter, LVPW: Left Ventricle Posterior Wall width of WT (blue circle) and *Psttm* (red circle) mice. s: systolic; d: diastolic. T-test shows that the left ventricle parameters between WT. Scale bar, (n=8) and *Psttm* (n=9) are statistically not different.

Table S1, related to Figures 1, 3, 5, 6 and 7. Sequences (5'-3') of primers used for mutagenesis, cloning of BiFC, chimeric FBXL and Y2H constructs, and CRISPR.

Flag-TCAP K26R F	CTTCTGGGCTGAGTGGAGAGACCTGACTCTGTCTA
Flag-TCAP K26R R	TAGACAGAGTCAGGTCTCTCCACTCAGCCCAGAAG
Flag-TCAP K98R F	CATCTTCACGCCCACCAGGGTGGGGGCCTCCAAGG
Flag-TCAP K98R R	CCTTGGAGGCCCCCACCCTGGTGGGCGTGAAGATG
Flag-TCAP K103R F	CAAGGTGGGGGCCTCCAGGGAGGAGCGCGAGGAGA
Flag-TCAP K103R R	TCTCCTCGCGCTCCTCCCTGGAGGCCCCCACCTTG
Flag-TCAP K138R F	CGTGGCTGAGATCACAAGGCAGCTTCCCCCTGTGG
Flag-TCAP K138R R	CCACAGGGGGAAGCTGCCTTGTGATCTCAGCCACG
Flag-TCAP K148R F	GTGCCAGTCAGCAGACCCGGGCCCCTG
Flag-TCAP K148R R	CAGGGGCCCGGGTCTGCTGACTGGCAC
Flag-/VenC- TCAP S157A F	CTGCGCCGTACCCTGGCTCGATCCATGTCTCAG
Flag-/VenC- TCAP S157A R	CTGAGACATGGATCGAGCCAGGGTACGGCGCAG
Flag-/VenC- TCAP S161A R	GCTCGATCCATGGCTCAGGAAGCTCAGAGA
Flag-/VenC- TCAP S161A R	TCTCTGAGCTTCCTGAGCCATGGATCGAGC
Flag-/VenN- FBXL21 T33A F	TCTTCCCTCCGCCAGGCCCATGCGCTCTCT
Flag-/VenN- FBXL21 T33A R	AGAGAGCGCATGGGCCTGGCGGAGGGAAGA
Flag-/VenN- FBXL21 S37A F	CAGGCCCATGCGCTCGCTGTCCTTCTGGAC
Flag-/VenN- FBXL21 S37A R	GTCCAGAAGGACAGCGAGCGCATGGGCCTG
Fbxl21-fbox-Fbxl3a-LRR F	TCCCTGATCTTTGGAGGAAGTTTGAATTTGAACTGA ATC A
Fbxl21-fbox-Fbxl3a-LRR R	TGATTCAGTTCAAATTCAAACTTCCTCCAAAGATCAGGGA
Fbxl3a-fbox-Fbxl21-LRR F	ACATGCCTGACTTGTGGCGATGTTTTGAGTTTGAGCTGAACCAAT
Fbxl3a-fbox-Fbxl21-LRR R	ATTGGTTCAGCTCAAACTCAAAACATCGCCACAAGTCAGGCATGT
Fbxl3a KpnI F	GGCGGTACCGATGAAACGAGGAGGAAGAGA
Fbxl3a XbaI R	GGCTCTAGATTACCAAGTAGGCATCATGT
KpnI-Fbxl21 F	GGCGGTACCGATGAAGAGGAATAATTTCTCT
XbaI-Fbxl21R	GGCTCTAGATTACCAGATGGGCATCACATCCGGA
Y2H-Fbxl3-F	GCGAATTCGAACTGAATCAGCCAGCG
Y2H-Fbxl3-R	GCGGATCCTTACCAAGTAGGCATCATGT
Y2H-Fbxl21-F	GCGAATTCTTTGAGCTGAACCAA
Y2H-Fbxl21-R	GCGGATCCGATGCCCATCTGGTAA
mFbxl21 CR(-) E4-F	CACCGTCTGGACTGGGGGACTTTA
mFbxl21 CR(-)E4-r	AAACTAAAGTCCCCCAGTCCAGAC
mFbxl21 CR(+) E4-F	CACCGTAGTGAAACAGCCCAAACG
mFbxl21 CR(+)E4-r	AAACCGTTTGGGCTGTTTCACTAC
